# Palladium-Catalyzed One-Pot Approach to 3-(Diarylmethylene)oxindoles from Propiolamidoaryl Triflate

**DOI:** 10.3390/molecules200814022

**Published:** 2015-08-03

**Authors:** Dahye Lee, Sunhwa Park, Yoseb Yu, Kye Jung Shin, Jae Hong Seo

**Affiliations:** Integrated Research Institute of Pharmaceutical Sciences, College of Pharmacy, The Catholic University of Korea, Bucheon-si, Gyeonggi-do 420-743, Korea; E-Mails: dahye8821@naver.com (D.L.); sunalovegs@catholic.ac.kr (S.P.); fbdytpq@catholic.ac.kr (Y.Y.); kyejung@catholic.ac.kr (K.J.S.)

**Keywords:** 3-(Diarylmethylene)oxindole, one-pot reaction, aryl triflate, palladium-catalyzed reaction

## Abstract

3-(Diarylmethylene)oxindoles have been synthesized from propiolamidoaryl triflate utilizing a palladium-catalyzed one-pot reaction consisting of three successive reactions: Sonogashira, Heck, and Suzuki-Miyaura. This method allows for the production of a complex skeleton of 3-(diarylmethylene)oxindole from propiolamidoaryl triflate using a commercially available aryl iodide and arylboronic acid in a simple and efficient way with moderate yield and stereoselectivity.

## 1. Introduction

**Figure 1:**
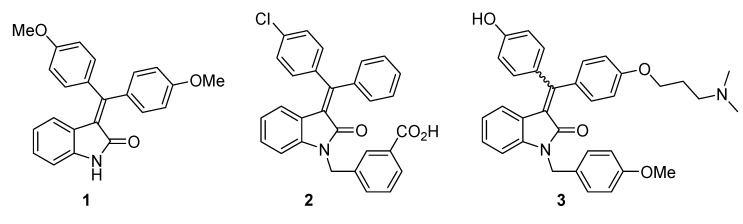
Biologically active 3-(diarylmethylene)oxindoles.

The oxindole scaffold, one of the major subfamilies of indoles, has been found in various biologically active natural products, therapeutics, and synthetic intermediates, which have attracted much interest from synthetic chemists to develop numerous methods for the synthesis of the unique scaffold [1,2,3]. Although synthetic studies on 3-(diarylmethylene)oxindoles are relatively rare, few biological activities have been reported (Figure 1). For example, TAS-301 (**1**) exhibits inhibitory activity of neointimal thickening following single balloon injury [4]. Recently, novel activities of the 3-(diarylmethylene)oxindoles have been revealed, such as AMPK activation of **2** [5] and anti-breast-cancer activity of **3** [6]. Due to these results, there is an increasing interest in the molecule’s structure. Over the last decade, several groups have reported efficient synthetic methods for 3-(diarylmethylene)oxindoles utilizing a metal-catalyzed tandem reaction [7,8,9,10,11,12,13,14]. However, more efficient synthetic methods for 3-(diarylmethylene)oxindoles are required.

**Scheme 1:**
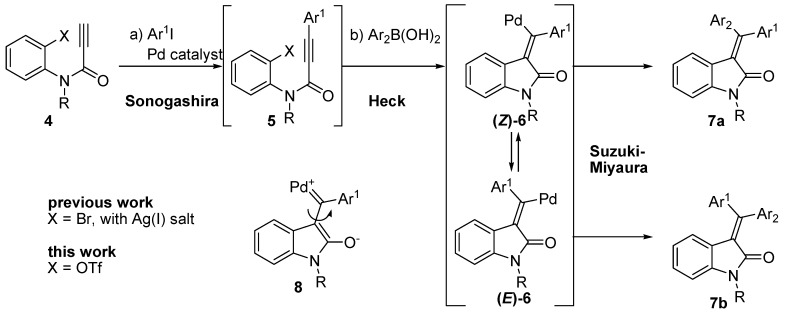
Palladium-catalyzed one-pot approach to 3-(diarylmethylene)oxindoles.

Due to our ongoing efforts to develop a novel domino reaction for the synthesis of biologically active heterocycles, we recently reported a palladium-catalyzed one-pot approach to 3-(diarylmethylene)oxindoles combining the Sonogashira, Heck, and Suzuki-Miyaura reactions (Scheme 1) [15]. In the one-pot reaction, a Sonogashira reaction of propiolamide **4** (X = Br) afforded the internal alkyne **5**, which was transformed into 3-(diarylmethylene)oxindole **7** by the addition of the corresponding arylboronic acid and elevation in reaction temperature. Unfortunately, the stereoselectivity for the newly formed olefin was low, yielding a mixture of *E-* and *Z-* isomers (**7a** and **7b**). This unexpected result could be rationalized by the formation of the zwitter ionic metal carbene intermediate **8**, with a rotatable σ-bond at the olefin position to facilitate interconversion between two vinylpalladium species, **(*Z*)-6** and **(*E*)-6** [16,17,18]. This assumption led us to add a silver (I) salt during the second step of the reaction to create **5** using the Heck reaction to proceed through the cationic pathway, which is likely to reduce the formation of the carbene intermediate **8** due to the pre-existence of the cationic charge of the vinyl palladium species **6**. To our delight, the addition of silver (I) salt dramatically increased stereoselectivity, strongly supporting the proposed carbene-intermediated *E*/*Z* isomerization mechanism [15].

The Heck reaction of the aryl triflate has been known to follow the cationic pathway due to complete dissociation of the triflate anion from the palladium center [19]. This fact prompted us to investigate the one-pot reaction with propiolamidoaryl triflate (**4**, X = OTf) as a substrate, with the expectation of identifying a solution to solve the low stereoselectivity of the arylbromo substrate (**4**, X = Br). Here, we describe optimization efforts for the one-pot reaction with aryl triflate **4** and substrate scope.

## 2. Results and Discussion

**Scheme 2:**
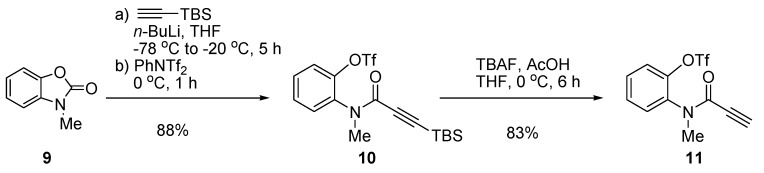
Synthesis of propiolamidophenyl triflate **11**.

The aryl triflate substrate **11** was prepared from a known benzoxazolone, **9** [20], using a modification of the Overman process (Scheme 2) [21]. The addition of TBS-acetylide to **9** gave the corresponding phenoxide, which was trapped by the triflating reagent to afford TBS-propiolamide **10** with a yield of 88%. Deprotection of the TBS group of **10** by TBAF under AcOH-buffered conditions gave rise to aryl triflate **11** with a yield of 83%.

When aryl triflate **11** was exposed to the previously optimized palladium-catalyzed one-pot conditions for the bromo-substrate [15], the desired 3-(diarylmethylene)oxindole **12** was formed with a yield of 64%, with 1,4-benzoxazine **13** as a by-product (7% yield) (entry 1) (Table 1). The unexpected formation of **13** was due to the base-sensitive and thermally-labile characteristics of triflates [22], causing decomposition of the triflate group of the Sonogashira adduct to the corresponding phenol compound, followed by 6-*endo*-*dig* cyclization [23,24], to give **13**. To investigate the effect of the base on the reaction, a number of bases were tested. Using KOAc as a base gave a similar yield of **12** and **13** (entry 1). The weak base, K_3_PO_4_, increased the yield of both **12** and **13** to 68% and 20%, respectively (entry 2). Interestingly, the reaction with Cs_2_CO_3_ was completed in a short time (2 h). However, the decomposition rate of the triflate group was much faster than the Heck/Suzuki-Miyaura domino reaction to give **13** as a major product with a 55% yield but a small amount of the desired oxindole, **12** (5% yield) (entry 4). K_2_CO_3_ also completed the reaction in 4 h, but **12** was obtained as the major product with a yield of 64% (entry 5). We decided to extend the reaction time to 3 h, expecting less decomposition of the triflate group. For NaOAc, changing the reaction time did not increase the yield of **12** (61% yield) (entry 6). In the case of K_3_PO_4_ as a base, the longer reaction time slightly increased the yield of **12** (72%), but substantially decreased the formation of **13** (5% yield, entry 7 *vs*. 20% yield, entry 3). The best result was obtained with K_2_CO_3_, which afforded **12** with a yield of 78% and a small amount of by-product, **13** (7% yield) (entry 8).

**Table 1:** Optimization of the one-pot reaction ^a^. 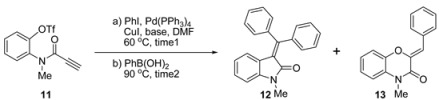

Entry	Base	Time 1 (h)	Time 2 (h)	Yield (%) ^b^	
				12	13
1	NaOAc	1	12	64	7
2	KOAc	1	12	58	10
3	K_3_PO_4_	1	12	68	20
4	Cs_2_CO_3_	1	2	5	55
5	K_2_CO_3_	1	4	64	29
6	NaOAc	3	12	61	7
7	K_3_PO_4_	3	3	72	5
8	K_2_CO_3_	3	2	78	7

^a^ reagents and conditions: PhI (1.1 equiv), Pd(PPh_3_)_4_ (10 mol %), CuI (5 mol %), base (3.0 equiv), DMF, 60 °C, Time 1; PhB (OH)_2_ (1.2 equiv), 90 °C, Time 2; ^b^ isolated yield.

After establishing optimal conditions, we investigated the substrate scope of the one-pot reaction (Table 2 First, we applied the reaction conditions to the synthesis of symmetrically substituted 3-(diarylmethylene)oxindoles. All reactions afforded the desired oxindoles, **14a**–**c**, with a moderate yield (42%–55% yields), regardless of substituents on aryliodide and arylboronic acid (entries 1–3). Comparing these results to that of the bromo substrate, **4** (X = Br) [15], product yields from triflate **11** were slightly lower, likely due to the formation of 1,4-benzoxazine (<10% yield). When phenyliodide was used as a Sonogashira coupling partner, the electronic character of the substituents on arylboronic acids affected the stereochemical outcome of the products (entries 4–6). Reaction with 4-methoxyphenylboronic acid afforded **14d** as a 1:1 mixture of *E*/*Z* isomers (entry 4). However, electron-withdrawing chloro- and nitro-substituents on the arylboronic acids increased *E*/*Z* stereoselectivity to 5:1 and 3:1, respectively (entries 5 and 6). Since the three above-mentioned reactions went through the same vinylpalladium intermediate, different stereochemical outcomes of these reactions suggested that formation of the *E*/*Z* mixture of the product was not only caused by *E*/*Z* isomerization of vinylpalladium species, **6**. One possible explanation for this result may be the isomerization between the two products under the reaction conditions. However, when pure each isomer was re-exposed to the reaction conditions (Pd(PPh_3_)_4_ (10 mol %), CuI (5 mol %), K_2_CO_3_ (3.0 equiv), DMF, 90 °C, 2 h) the interconversion between two *E/Z* isomeric products was less than 20%. This result ruled out isomerization of the product from the main contributor of low stereoselectivity [15]. However, since the reactions of the bromo substrate with the same combination of phenyliodide and arylboronic acids showed no selectivity on the *E*/*Z* isomers [15], enhanced stereoselectivity by the triflate substrate strongly supports that isomerization could be reduced under the cationic pathway. Substituents of aryliodides also exerted an effect on the stereoselectivity of the products. The reaction with 4-mehoxyphenyl iodide resulted in a low *E*/*Z* ratio (1.5:1) (entry 7). 4-Chloro- and 4-nitro-phenyl iodide both showed good stereoselectivity (*E*/*Z* = 1:5 and 1:12, respectively) (entries 8 and 9). The stereoselectivity of the last two results are higher than those of the bromo substrate, which proved the usefulness of the triflate substrate for stereoselective synthesis of unsymmetrically substituted 3-(diarylmethylene)oxindole.

**Table 2:** Substrate scope of the one-pot reaction ^a^. 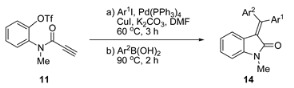

Entry	Ar^1^	Ar^2^	14	Yield (%) ^b^	*E*/*Z* Ratio ^c^	Bromo Substrate (Ref. [15])	
						Yield (%) ^b^	*E*/*Z* Ratio ^c^
1	4-MeOPh	4-MeOPh	**14a**	55	-	85	-
2	4-ClPh	4-ClPh	**14b**	54	-	80	-
3	4-NO_2_Ph	4-NO_2_Ph	**14c**	42	-	67 ^d^	-
4	Ph	4-MeOPh	**14d**	58	1:1	52	1:1
5	Ph	4-ClPh	**14e**	65	5:1	57	1:1
6	Ph	4-NO_2_Ph	**14f**	59	3:1	52	1:1
7	4-MeOPh	Ph	**14d**	62	1.5:1	70	1.6:1
8	4-ClPh	Ph	**14e**	66	1:5	77	1:3.4
9	4-NO_2_Ph	Ph	**14f**	62 ^d^	1:12	73 ^d^	1:10

^a^ reagents and conditions: PhI (1.1 equiv), Pd(PPh_3_)_4_ (10 mol %), CuI (5 mol %), K_2_CO_3_ (3.0 equiv), DMF, 60 °C, 3 h; PhB(OH)_2_ (1.2 equiv), 90 °C, 2 h; ^b^ isolated yield; ^c^ ratio between isolated *E*/*Z* isomers; ^d^ Heck/Suzuki-Miyaura reaction was conducted at 110 °C.

## 3. Experimental Section 

### 3.1. General Information

All reactions were performed under an argon atmosphere with dry solvents, unless otherwise stated. Dry diethyl ether (Et_2_O), tetrahydrofuran (THF), acetonitrile (MeCN), toluene, and methylene chloride (CH_2_Cl_2_) were obtained from Ultimate Solvent Purification System (JC Meyer Solvent System, Laguna Beach, CA, USA). Other dry solvents were purchased as anhydrous grade. All commercially available reagents were purchased and used without further purification. Reactions were monitored by thin-layer chromatography (TLC) on silica gel plates (Merck TLC Silica Gel 60 F_254_) using UV light, PMA (an ethanolic solution of phosphomolybdic acid) or ANIS (an ethanolic solution of *para*-anisaldehyde) as visualizing agent. Purification of products was conducted by column chromatography through silica gel 60 (0.060–0.200 mm). Melting points of all solid compounds were determined by Buchi M-565 (BÜCHI Labortechnik AG, Flawil, Switzerland). NMR spectra were obtained on Bruker AVANCE III 500 MHz (Bruker Corporation, Billerica, MA, USA) using residual undeuterated solvent or TMS (tetramethylsilane) as an internal reference. IR spectra were recorded on a Jasco P-2000 FT-IR spectrometer (JASCO, Easton, MD, USA). High-resolution mass spectra (HR-MS) were recorded on an Agilent 6530 Q-TOF mass spectrometer (Agilent Tchnology, Santa Clara, CA, USA) using ESI (electrospray ionization) or a JEOL JMS-700 (JEOL, Tokyo, Japan) using EI (electron impact). Copies of NMR spectra of all compounds are available in the Supplementary Materials (Supplementary Figures S1–S17).

### 3.2. Preparation of Propiolamidoaryl Triflate **11**

*2-(3-(Tert-butyldimethylsilyl)-N-methylpropiolamido)phenyl trifluoromethanesulfonate* (**10**): To a stirred solution of TBS-acetylene (1.30 mL, 6.96 mmol) in THF (30 mL) was added *n*-BuLi (2.0 M in hexanes, 3.2 mL, 6.4 mmol) at −78 °C. After 1 h, a solution of *N*-methyl-2-benzoxzolinone (**9**) [20] (881 mg, 5.91 mmol) in THF (15 mL) was added dropwise to the reaction mixture. Then, the reaction temperature was gradually raised to −20 °C. After 4 h stirring at −20 °C, to the mixture was added a solution of *N*-phenylbis(trifluoromethansulfonimide) (3.2 g, 9.0 mmol) in THF (15 mL). The reaction mixture was warmed to 0 °C and stirred for 1 h. Then, the mixture was diluted with sat. aq. NH_4_Cl (30 mL) and EtOAc (300 mL). The organic layer was separated, dried (Na_2_SO_4_), filtered and concentrated under reduced pressure. The crude residue was purified by column chromatography (silica gel, hexanes:EtOAc 4:1) to afford TBS-propiolamide **10** (2.20 g, 88% yield) as a pale yellow oil. **10**: *R*_f_ = 0.16 (silicagel, hexanes:EtOAc 4:1); IR (film) 3726, 2984, 1652, 1496, 1423, 1361, 1308, 1212, 1140, 940, 882, 827, 778 cm^−1^; ^1^H-NMR (500 MHz, CDCl_3,_ 3:1 atropisomeric mixture): δ = 7.47–7.31 (m, 4H, *major and minor*), 3.56 (s, 3H, *minor*), 3.28 (s, 3H, *major*), 0.99 (s, 9H, *minor*), 0.68 (s, 9H, *major*), 0.20 (s, 6H, *minor*), −0.08 (d, *J* = 3.5 Hz, 6H, *major*) ppm; ^13^C-NMR (125 MHz, CDCl_3_, *major peaks*) δ = 153.7, 145.5, 136.0, 131.0, 130.1, 129.3, 122.8, 118.6 (q, ^1^*J*_C-F_ = 320.1 Hz), 97.3, 96.5, 36.0, 25.8, 16.3, −5.4 ppm; HRMS (FAB): calcd for C_17_H_2__3_F_3_NO_4_SSi [M + H]^+^: 422.1069, found 422.1073.

*2-(N-methylpropiolamido)phenyl trifluoromethanesulfonate* (**11**): To a stirred solution of TBS-propiolamide **10** (912 mg, 3.15 mmol) in THF (30 mL) was added TBAF (1.0 M in THF, 4.15 mL, 4.15 mmol) and AcOH (0.18 mL, 3.14 mmol) at 0 °C. After 24 h, the reaction mixture was diluted with sat. aq. NH_4_Cl (30 mL) and EtOAc (100 mL). The organic layer was separated, dried (Na_2_SO_4_), filtered and concentrated under reduced pressure. The crude residue was purified by column chromatography (silica gel, hexanes:EtOAc 4:1) to afford propiolamide **11** (457 mg, 83% yield) as white solid. **11**: mp = 70.1 °C (uncorrect); *R*_f_ = 0.38 (silicagel, hexanes:EtOAc 2:1); IR (film) 3727, 3229, 2923, 2852, 2109, 1778, 1652, 1494, 1421, 1368, 1309, 1213, 1138, 1092, 880, 769, 737, 681 cm^−1^; ^1^H-NMR (500 MHz, CDCl_3,_ 3:1 atropisomeric mixture): δ = 7.52–7.41 (m, 4H, *major*,2H,*minor*), 7.38–7.36 (m, 2H, *minor*), 3.59 (s, 3H, *minor*), 3.31 (s, 3H, *major*), 3.27 (s, 1H, *minor*), 2.79 (s, 1H, *major*) ppm; ^13^C-NMR (125 MHz, CDCl_3_, *major peaks*): δ = 153.1, 145.4, 135.5, 130.8, 130.5, 129.4, 122.8, 118.6 (q, ^1^*J*_C-F_ = 318.6 Hz), 79.4, 75.6, 36.1 ppm; HRMS (EI): Calcd for C_11_H_8_F_3_NO_4_S [M]^+^: 307.0126, found 307.0121.

### 3.3. General Procedure for Palladium-Catalyzed One-Pot Reaction

To a stirred solution of propiolamide **11** (0.5 mmol, 1.0 equiv) in DMF (5 mL) were added the corresponding aryl iodide (0.55 mmol, 1.1 equiv), CuI (0.025 mmol, 5 mol %), K_2_CO_3_ (1.5 mmol, 3.0 equiv) and Pd(PPh_3_)_4_ (0.05 mmol, 10 mol %) at 25 °C. The reaction mixture was stirred at 60 °C for 3 h. Then, the corresponding aryl boronic acid (0.6 mmol, 1.2 equiv) was quickly added to the mixture. Reaction temperature was raised to 90 °C or 110 °C. After 2 h stirring at the indicated temperature, the mixture was cooled to 25 °C and diluted with EtOAc (200 mL). The organic layer was washed with H_2_O (30 mL × 3) and brine (30 mL), then dried (Na_2_SO_4_), filtered and concentrated under reduced pressure. The crude residue was purified by column chromatography (silica gel, hexane:EtOAc) to yield 3-(diarylmethylene)oxindoles (**12** and **14a**–**f**) and 1,4-benzoxazine **13**.

*3-(Diphenylmethylene)-1-methylindolin-2-one* (**12**): Yellow solid, mp = 165.9 °C (lit. [13], 153.2–154.9 °C); *R*_f_ = 0.33 (silica gel, hexanes:EtOAc 4:1); IR (film) 3054, 2923, 1700, 1606, 1469 cm^−1^; ^1^H-NMR (500 MHz, CDCl_3_): δ = 7.45–7.41 (m, 3H), 7.37–7.32 (m, 7H), 7.17 (td, *J* = 7.5, 1.0 Hz, 1H), 6.76 (d, *J* = 8.0 Hz, 1H), 6.68 (td, *J* = 7.5, 1.0 Hz, 1H), 6.42 (dd, *J* = 7.5, 1.0 Hz, 1H), 3.21 (s, 3H) ppm; ^13^C-NMR (125 MHz, CDCl_3_): δ = 167.0, 154.7, 143.5, 141.5, 140.1, 130.1, 129.5, 129.3, 129.2, 129.1, 128.9, 128.0, 124.4, 123.4, 123.3, 121.5, 107.8, 26.0 ppm; HRMS (ESI-TOF): calcd for C_22_H_17_ NO [M + H]^+^: 312.1388, found 312.1394.

*(Z)-2-Benzylidene-4-methyl-2H-benzo[b][1,4]oxazin-3(4H)-one* (**13**): White solid; mp = 155.1 °C (lit. [25], 155–156 °C); *R*_f_ = 0.60 (silicagel, CH_2_Cl_2_); IR (film) 3727, 2923, 2853, 1668, 1630, 1504, 1422, 1381, 1288, 1167, 1127, 1039, 743, 686 cm^−1^; ^1^H-NMR (500 MHz, CDCl_3_): δ = 7.85 (d, *J* = 7.5 Hz, 2H), 7.41 (t, *J* = 7.5 Hz, 2H), 7.31 (t, *J* = 7.3 Hz, 1H), 7.20–7.18 (m, 1H), 7.10–7.07 (m, 2H), 7.02–7.00 (m, 1H), 6.96 (s, 1H), 3.48 (s, 3H) ppm; ^13^C-NMR (125 MHz, CDCl_3_): δ = 157.4, 142.0, 141.2, 133.9, 130.1, 128.7, 128.3, 127.6, 123.9, 123.4, 116.1, 114.5, 113.0, 28.8 ppm; HRMS (EI): calcd for C_16_H_13_NO_2_ [M]^+^: 251.0946, found 251.0947.

*3-(bis(4-Methoxyphenyl)methylene)-1-methylindolin-2-one* (**14a**): Brown solid; mp = 192.2 °C; *R*_f_ = 0.2 (silica gel, hexanes:EtOAc 4:1); IR (film) 3017, 1691, 1603, 1251 cm^−1^; ^1^H-NMR (500 MHz, CDCl_3_): δ = 7.29–7.24 (m, 4H), 7.14 (td, *J* = 8.0, 1.5 Hz, 1H), 6.93 (d, *J* = 9.0 Hz, 2H), 6.88 (d, *J* = 9.0 Hz, 2H), 6.77 (d, *J* = 8.0 Hz, 1H), 6.70 (td, *J* = 8.0, 1.5 Hz, 1H), 6.57 (dd, *J* = 7.5, 0.5 Hz, 1H), 3.88 (s, 3H), 3.84 (s, 3H), 3.22 (s, 3H) ppm; ^13^C-NMR (125 MHz, CDCl_3_): δ = 167.3, 161.0, 160.8, 155.1, 142.8, 133.9, 132.9, 132.4, 132.1, 128.0, 124.3, 122.7, 122.5, 121.3, 114.2, 113.2, 107.7, 55.5, 55.4, 26.0 ppm; HRMS (ESI-TOF): calcd for C_24_H_21_ NO_3_ [M + H]^+^: 372.1600, found 372.1611.

*3-(bis(4-Chlorophenyl)methylene)-1-methylindolin-2-one* (**14b**): Yellow solid; mp = 134.4 °C; *R*_f_ = 0.33 (silica gel, hexanes:EtOAc 4:1); IR (film) 3054, 2927, 1699, 1606, 1486, 1089 cm^–1^; ^1^H-NMR (500 MHz, CDCl_3_): δ = 7.41 (dd, *J* = 7.0, 2.0 Hz, 2H), 7.33 (dd, *J* = 7.0, 2.0 Hz, 2H), 7.26–7.20 (m, 5H), 6.78 (d, *J* = 8.0 Hz, 1H), 6.73 (td, *J* = 7.5, 1.0 Hz, 1H), 6.52 (d, *J* = 7.5 Hz, 1H), 3.20 (s, 3H) ppm; ^13^C-NMR (125 MHz, CDCl_3_): δ = 166.7, 151.4, 143.5, 139.3, 138.0, 135.7, 135.6, 131.7, 131.1, 129.5, 129.4, 128.3, 125.1, 123.2, 122.8, 121.7, 108.1, 26.0 ppm; HRMS (ESI-TOF): calcd for C_22_H_15_Cl_2_ NO [M + H]^+^: 380.0609, found 380.0612.

*3-(bis(4-Nitrophenyl)methylene)-1-methylindolin-2-one* (**14c**): Brown solid; mp = 244.5 °C; *R*_f_ = 0.21 (silica gel, hexanes:EtOAc 3:1); IR (film) 3073, 1703, 1601, 1517, 1486, 1344, 1098 cm^−1^; ^1^H-NMR (500 MHz, CDCl_3_): δ = 8.34 (dd, *J* = 7.0, 2.0 Hz, 2H), 8.24 (dd, *J* = 7.0, 2.0 Hz, 2H), 7.56 (dd, *J* = 7.0, 2.0 Hz, 2H), 7.48 (dd, *J* = 7.0, 2.0 Hz, 2H), 7.29 (dd, *J* = 7.5, 1.0 Hz, 1H), 6.83 (d, *J* = 8.0 Hz, 1H), 6.76 (td, *J* = 8.0, 1.0 Hz, 1H), 6.45 (d, *J* = 7.5, 1H), 3.20 (s, 3H) ppm; ^13^C-NMR (125 MHz, CDCl_3_): δ = 166.0, 148.4, 148.1, 147.1, 146.3, 145.6, 144.3, 130.9, 130.6, 130.4, 127.5, 124.8, 123.63, 123.61, 122.2, 121.4, 108.7, 26.1 ppm; HRMS (ESI-TOF): calcd for C_22_H_15_N_3_O_5_ [M + H]^+^: 402.1092, found 402.1097.

*(Z)-3-((4-Methoxyphenyl)(phenyl)methylene)-1-methylindolin-2-one* (***Z*-14d**): Yellow solid; mp = 153.0 °C (lit. [26], 163.8-164.3 °C); *R*_f_ = 0.24 (silica gel, hexanes:EtOAc 4:1); IR (film) 2931, 1696, 1604, 1250 cm^−1^; ^1^H-NMR (500 MHz, CDCl_3_): δ = 7.46–7.40 (m, 3H), 7.32–7.29 (m, 4H), 7.15 (t, *J* = 13.0 Hz, 1H), 6.88 (dd, *J* = 7.0, 2.0 Hz, 2H), 6.76 (d, *J* = 8.0 Hz, 1H), 6.66 (t, *J* = 7.5 Hz, 1H), 6.32 (d, *J* = 7.5 Hz, 1H), 3.84 (s, 3H), 3.23 (s, 3H) ppm; ^13^C-NMR (125 MHz, CDCl_3_): δ = 167.1, 161.0, 154.9, 143.0, 141.7, 132.7, 131.9, 129.9, 129.3, 129.0, 128.4, 123.9, 123.0, 121.4, 113.2, 107.7, 55.4, 26.0 ppm; HRMS (ESI-TOF): Calcd for C_23_H_19_ NO_2_ [M + H]^+^: 342.1494, found 342.1503.

*(E)-3-((4-Methoxyphenyl)(phenyl)methylene)-1-methylindolin-2-one* (***E*-14d**): Yellow solid; mp = 162.7 °C (lit. [13], 163.8-164.3 °C); *R*_f_ = 0.26 (silica gel, hexanes:EtOAc 4:1); IR (film) 3053, 2927, 1696, 1603, 1508, 1469, 1250 cm^−1^; ^1^H-NMR (500 MHz, CDCl_3_): δ = 7.39–7.25 (m, 7H), 7.17 (td, *J* = 7.5, 1.5 Hz, 1H), 6.93 (dd, *J* = 7.0, 2.0 Hz, 2H), 6.78–6.67 (m, 3H), 3.87 (s, 3H), 3.20 (s, 3H) ppm; ^13^C-NMR (125 MHz, CDCl_3_): δ = 167.1, 160.8, 154.9, 143.3, 140.6, 133.6, 132.7, 131.7, 130.5, 129.3, 129.0, 128.6, 127.9, 122.9, 121.4, 114.3, 107.8, 55.5, 26.0 ppm; HRMS (ESI-TOF): calcd for C_23_H_19_ NO_2_ [M + H]^+^: 342.1494, found 342.1498.

*(Z)-3-((4-Chlorophenyl)(phenyl)methylene)-1-methylindolin-2-one* (***Z*-14e**): Yellow solid; mp = 59.0 °C: *R*_f_ = 0.37 (silica gel, hexanes:EtOAc 4:1); IR (film) 3054, 1698, 1606, 1486, 1335, 1090 cm^−1^; ^1^H-NMR (500 MHz, CDCl_3_): δ = 7.48–7.43 (m, 3H), 7.35–7.27 (m, 6H), 7.17 (td, *J* = 7.5, 1.0 Hz, 1H), 6.77 (d, *J* = 8.0 Hz, 1H), 6.68 (td, *J* = 7.5, 1.0 Hz, 1H), 6.42 (dd, *J* = 7.5, 0.5 Hz, 1H), 3.20 (s, 3H) ppm; ^13^C-NMR (125 MHz, CDCl_3_): δ = 166.9, 153.0, 143.5, 141.0, 138.4, 135.3, 131.6, 129.51, 129.50, 129.2, 129.1, 128.2, 124.7, 123.4, 123.2, 121.6, 107.9, 26.0 ppm; HRMS (EI): Calcd for C_22_H_16_ClNO [M]^+^: 345.0920, found 345.0916.

*(E)-3-((4-Chlorophenyl)(phenyl)methylene)-1-methylindolin-2-one* (***E*-14e**): Yellow solid; mp = 54.8 °C; *R*_f_ = 0.35 (silica gel, hexanes:EtOAc 4:1); ^1^H-NMR (500 MHz, CDCl_3_): δ = 7.41–7.18 (m, 9H), 7.19 (td, *J* = 7.5, 1.0 Hz, 1H), 6.78 (d, *J* = 7.5 Hz, 1H), 6.73 (td, *J* = 7.5, 1.0 Hz, 1H), 6.53 (dd, *J* = 7.5, 0.5 Hz, 1H), 3.20 (s, 3H) ppm; ^13^C-NMR (125 MHz, CDCl_3_): δ = 166.8, 153.0, 143.6, 139.8, 139.7, 135.5, 131.1, 130.2, 129.42, 129.37, 129.2, 128.1, 124.7, 123.2, 123.0, 121.6, 108.0, 26.0 ppm; HRMS (EI): Calcd for C_22_H_16_ClNO [M]^+^: 345.0920, found 345.0919.

*(Z)-1-Methyl-3-((4-nitrophenyl)(phenyl)methylene)indolin-2-one* (***Z*-14f**): Yellow solid; mp = 186.6 °C; *R*_f_ = 0.28 (silica gel, hexanes:EtOAc 4:1); IR (film) 3057, 1699, 1603, 1510, 1342 cm^−1^; ^1^H-NMR (500 MHz, CDCl_3_): δ = 8.22 (dd, *J* = 7.0, 1.5 Hz, 2H), 7.50–7.44 (m, 5H), 7.32 (dd, *J* = 8.0, 2.0 Hz, 2H), 7.22 (t, *J* = 7.5 Hz, 1H), 6.80 (d, *J* = 8.0 Hz, 1H), 6.73 (t, *J* = 7.75 Hz, 1H), 6.52 (d, *J* = 8.0 Hz, 1H), 3.20 (s, 3H) ppm; ^13^C-NMR (125 MHz, CDCl_3_): δ = 166.6, 150.8, 147.8, 147.0, 143.9, 140.0, 130.7, 129.9, 129.8, 129.4, 129.2, 126.1, 123.7, 123.4, 122.4, 121.9, 108.2, 26.0 ppm; HRMS (ESI-TOF): Calcd for C_22_H_16_ N_2_O_3_ [M + H]^+^: 357.1239, found 357.1249.

*(E)-1-Methyl-3-((4-nitrophenyl)(phenyl)methylene)indolin-2-one* (***E*-14f**): Yellow solid; mp = 201.2 °C (lit. [13], 198.8–200.8 °C); *R*_f_ = 0.25 (silica gel, hexanes:EtOAc 4:1); IR (film) 3056, 1701, 1604, 1519, 1469, 1345 cm^−1^; ^1^H-NMR (500 MHz, CDCl_3_): δ = 8.30 (t, *J* = 4.25 Hz, 2H), 7.54 (d, *J* = 9.0 Hz, 2H), 7.41–7.21 (m, 6H), 6.80 (d, *J* = 8.0 Hz, 1H), 6.71 (t, *J* = 7.75 Hz, 1H), 6.36 (d, *J* = 7.5 Hz, 1H), 3.21 (s, 3H) ppm; ^13^C-NMR (125 MHz, CDCl_3_): δ = 166.4, 150.9, 148.2, 147.9, 143.9, 138.8, 130.6, 130.0, 129.9, 129.7, 128.3, 125.7, 124.4, 123.3, 122.4, 121.8, 108.3, 26.1 ppm; HRMS (EI): calcd for C_22_H_16_N_2_O_3_ [M]^+^: 356.1161, found 356.1161.

## 4. Conclusions

In conclusion, we developed an efficient method for the synthesis of 3-(diarylmethylene)oxindoles from propiolamidoaryl triflate. Aryl triflate showed greater *E*/*Z* selectivity than those of the bromo substrate, which proved itself as another solution for low stereoselectivity of the palladium-catalyzed one-pot reaction, and supports the previously proposed isomerization mechanism of vinylpalladium species via a zwitter ionic metal carbene intermediate.

## Supplementary Materials

Supplementary materials can be accessed at: http://www.mdpi.com/1420-3049/20/08/14022/s1.
