# Supplementary material for: Palladium-Catalyzed One-Pot Approach to 3-(Diarylmethylene)oxindoles from Propiolamidoaryl Triflate
Source: Molecules. 2015 Aug 3;20(8):14022–32. doi: 10.3390/molecules200814022 (PMC6331821; doi:10.3390/molecules200814022)
Supplement: Supplementary file 1 [file molecules-20-14022-s001.pdf]

Supplementary

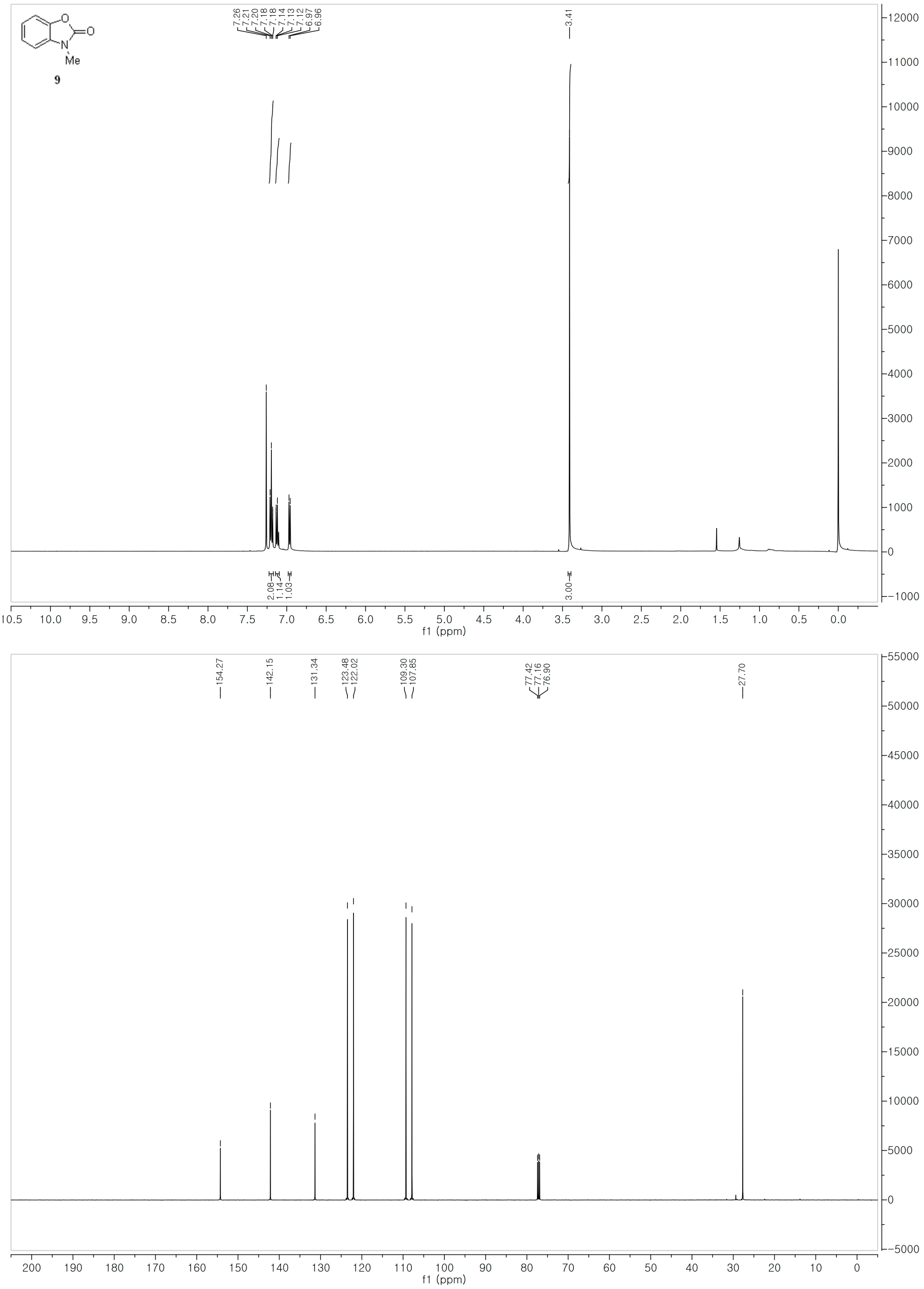

Figure S1. ^{1}H and ^{13}C spectrum of 3-methylbenzo[*d*]oxazol-2(3*H*)-one (**9**).

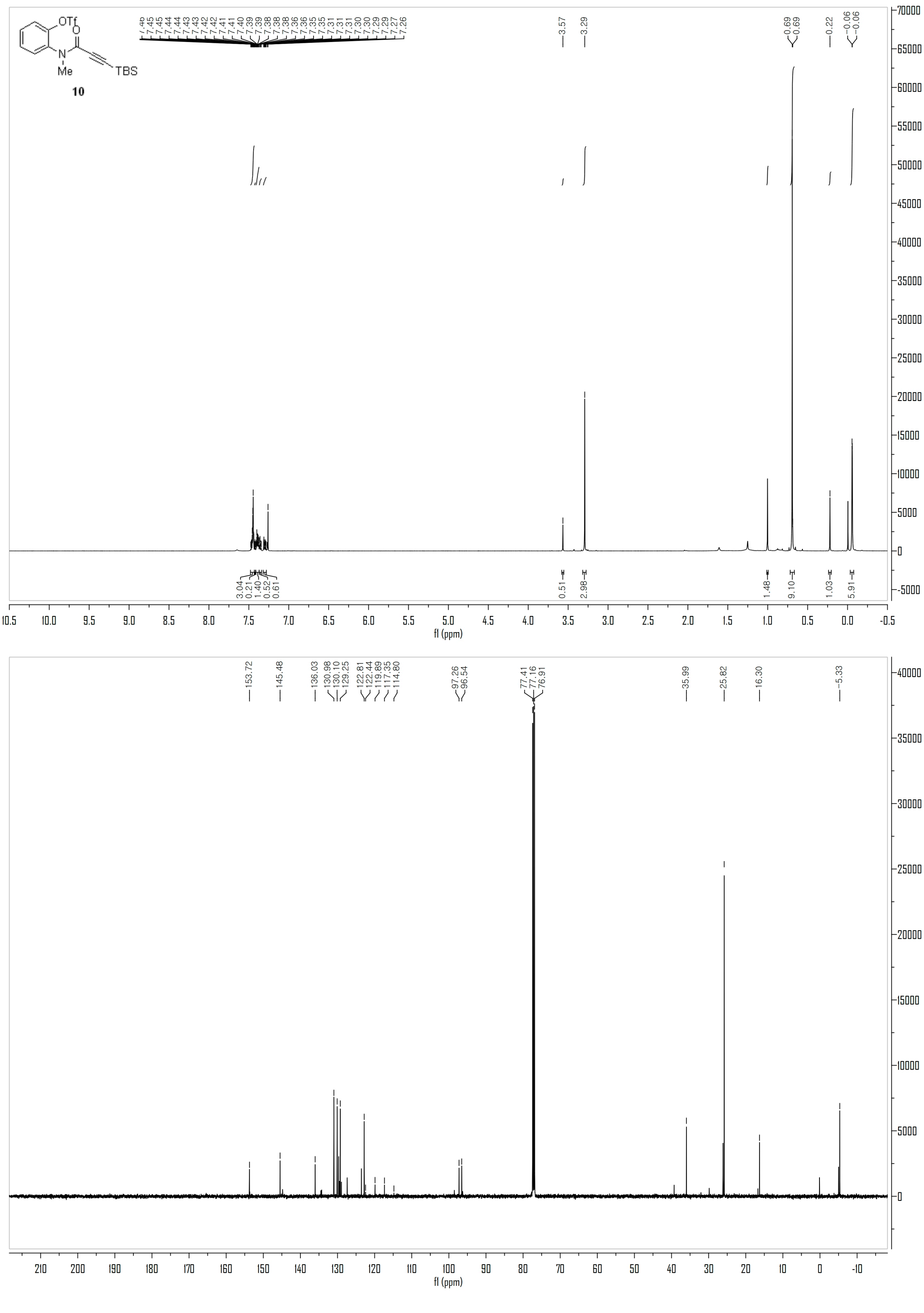

Figure S2. ^{1}H and ^{13}C spectrum of 2-(3-(*tert*-butyldimethylsilyl)-*N*-methylpropiolamido) phenyl trifluoromethanesulfonate (**10**).

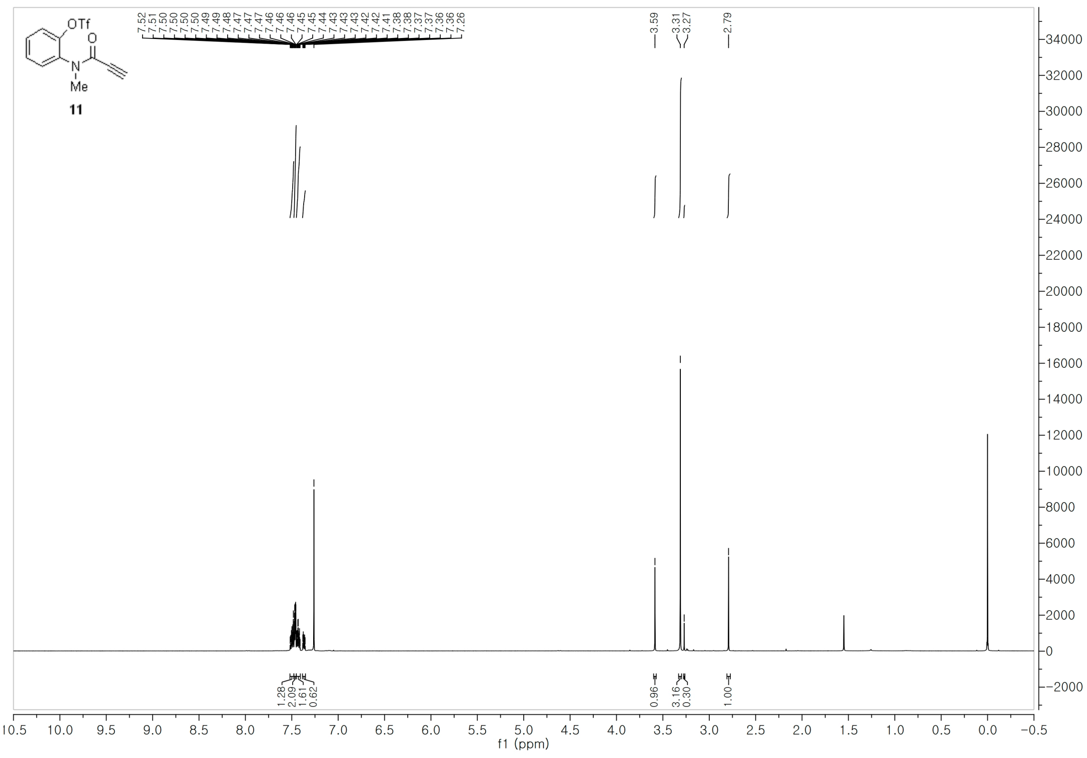

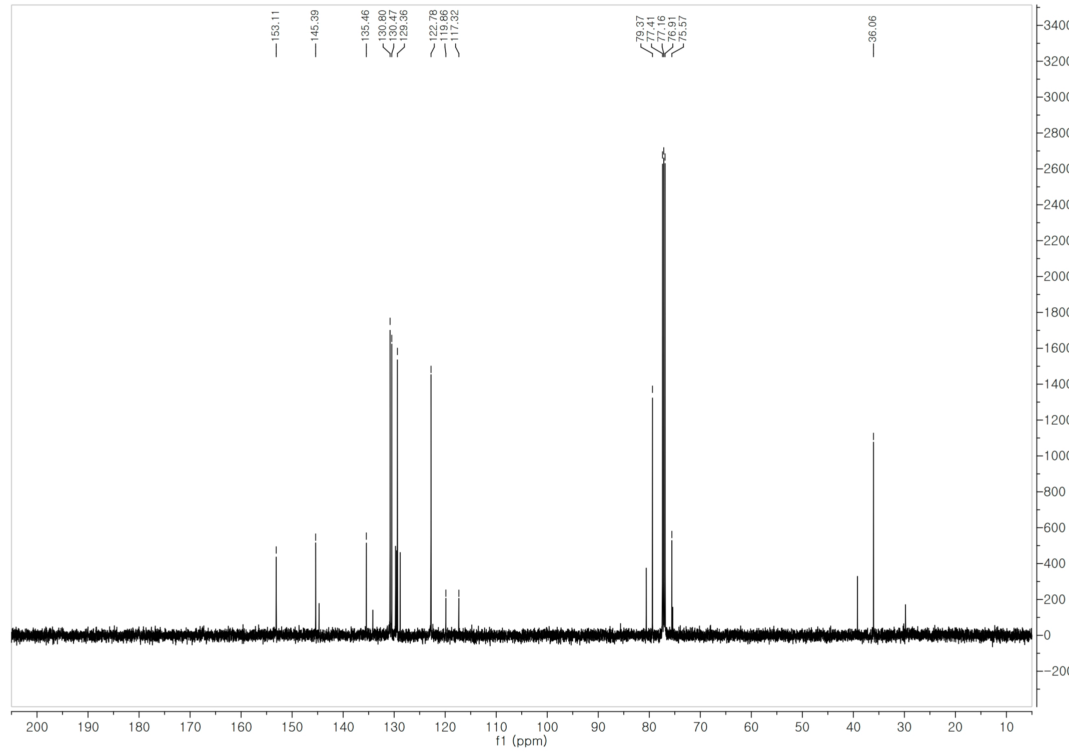

Figure S3. ^{1}H and ^{13}C spectrum of 2-(*N*-methylpropiolamido)phenyl trifluoromethanesulfonate (**11**).

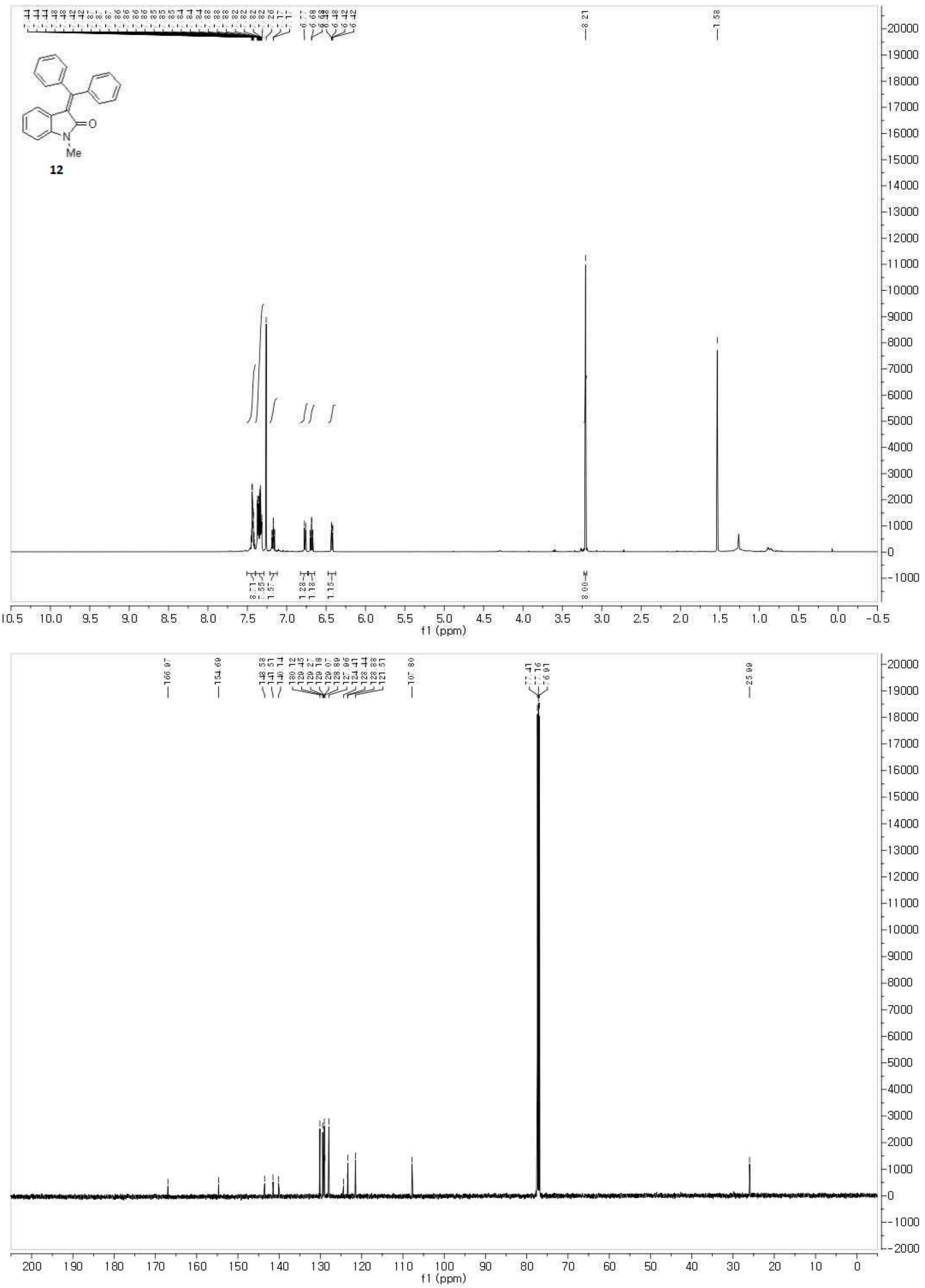

Figure S4. ^{1}H and ^{13}C spectrum of 3-(diphenylmethylene)-1-methylindolin-2-one (**12**).

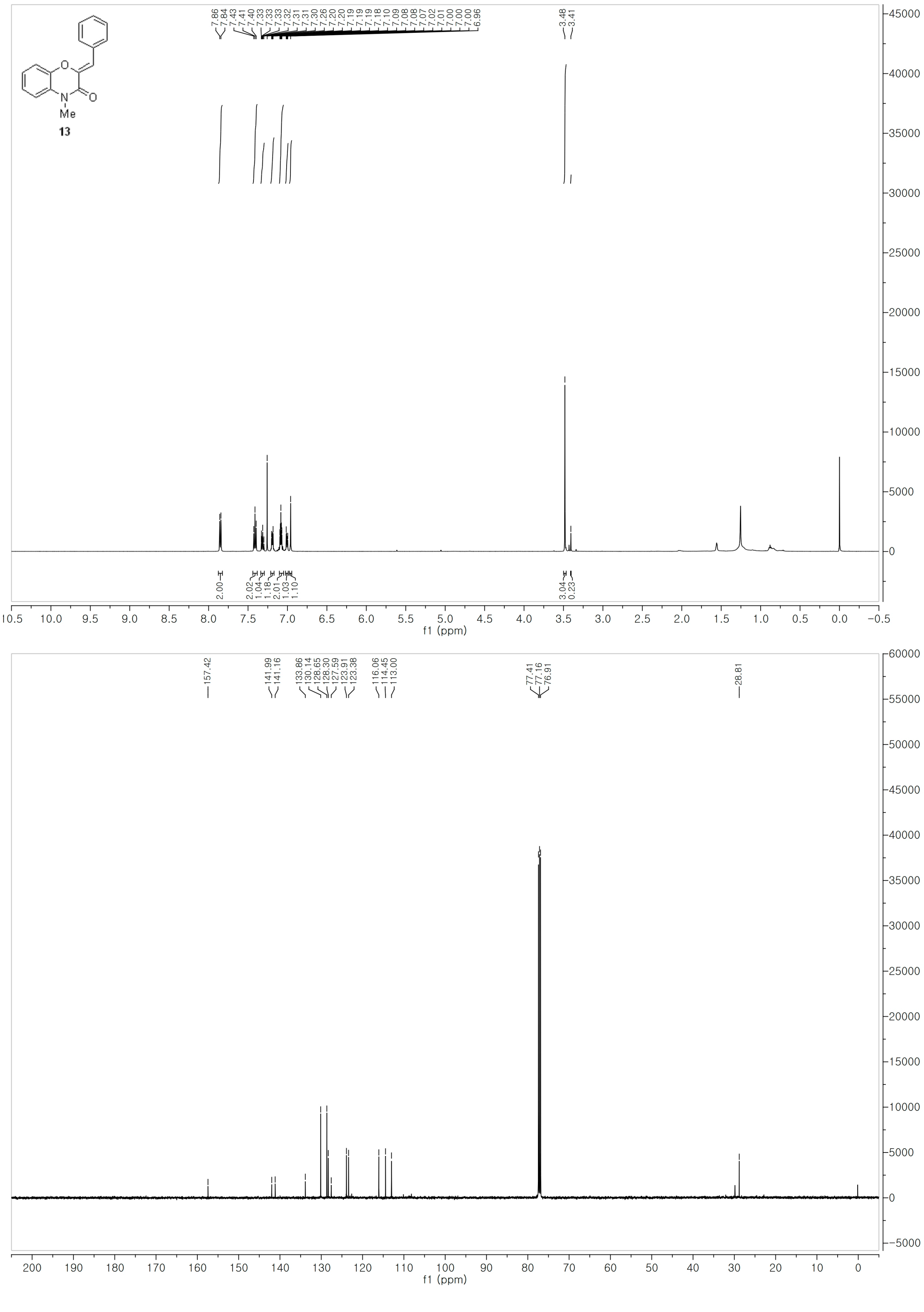

Figure S5. ^{1}H and ^{13}C spectrum of (*Z*)-2-benzylidene-4-methyl-2*H*-benzo[*b*][1,4]oxazin-3(4*H*)-one (**13**).

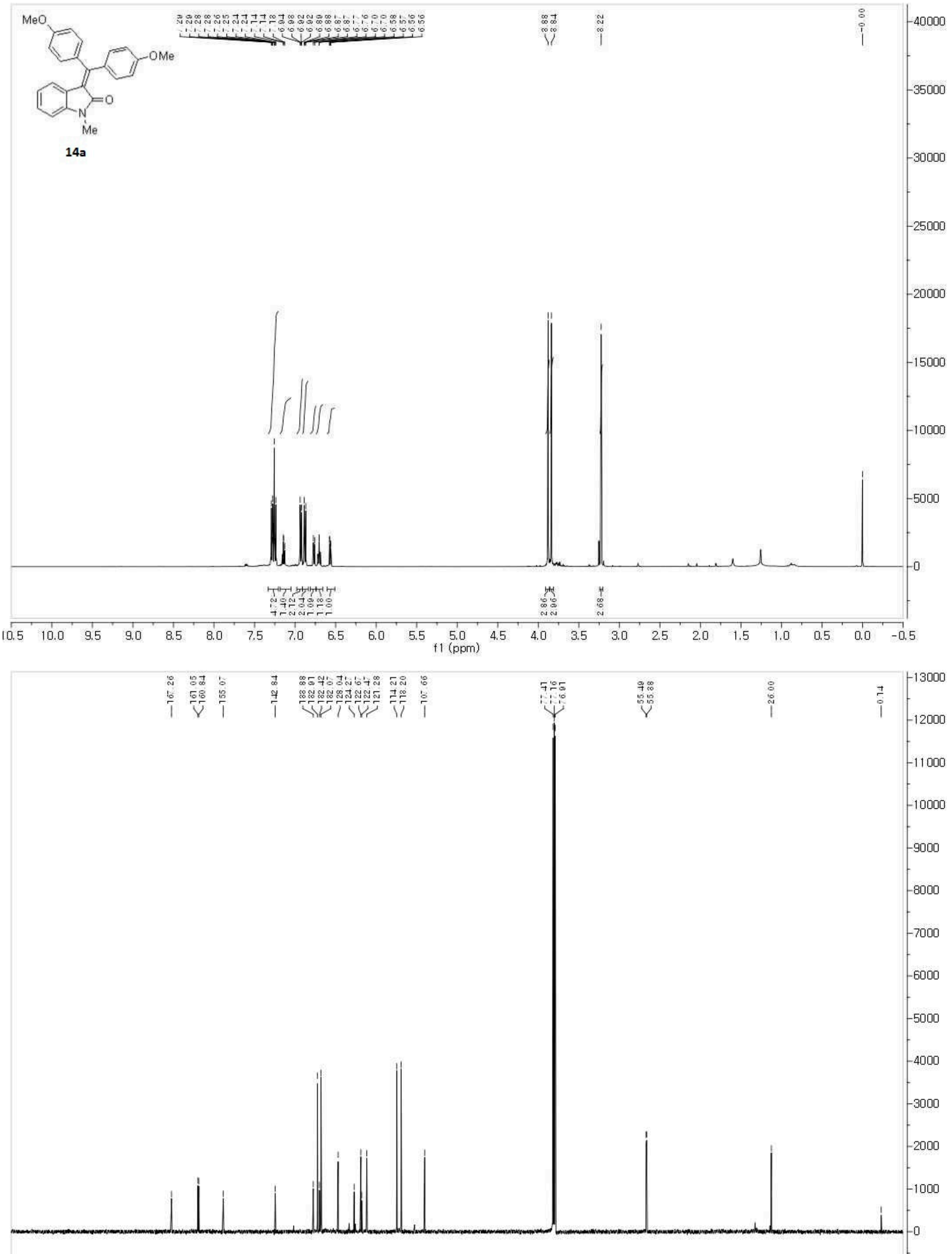

Figure S6. ^{1}H and ^{13}C spectrum of 3-(bis(4-methoxyphenyl)methylene)-1-methylindolin-2-one (**14a**).

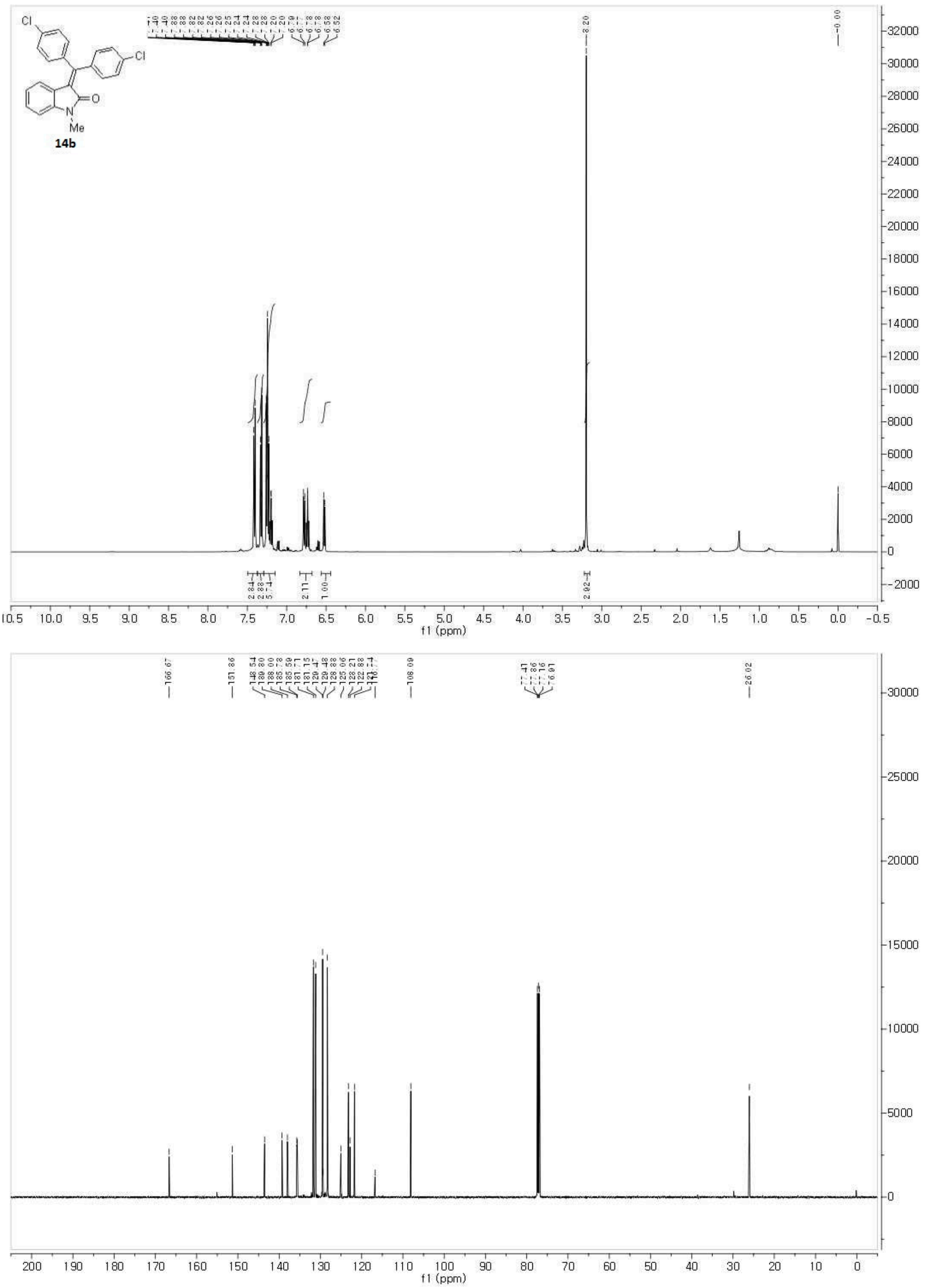

Figure S7. ^{1}H and ^{13}C spectrum of 3-(bis(4-chlorophenyl)methylene)-1-methylindolin-2-one (**14b**).

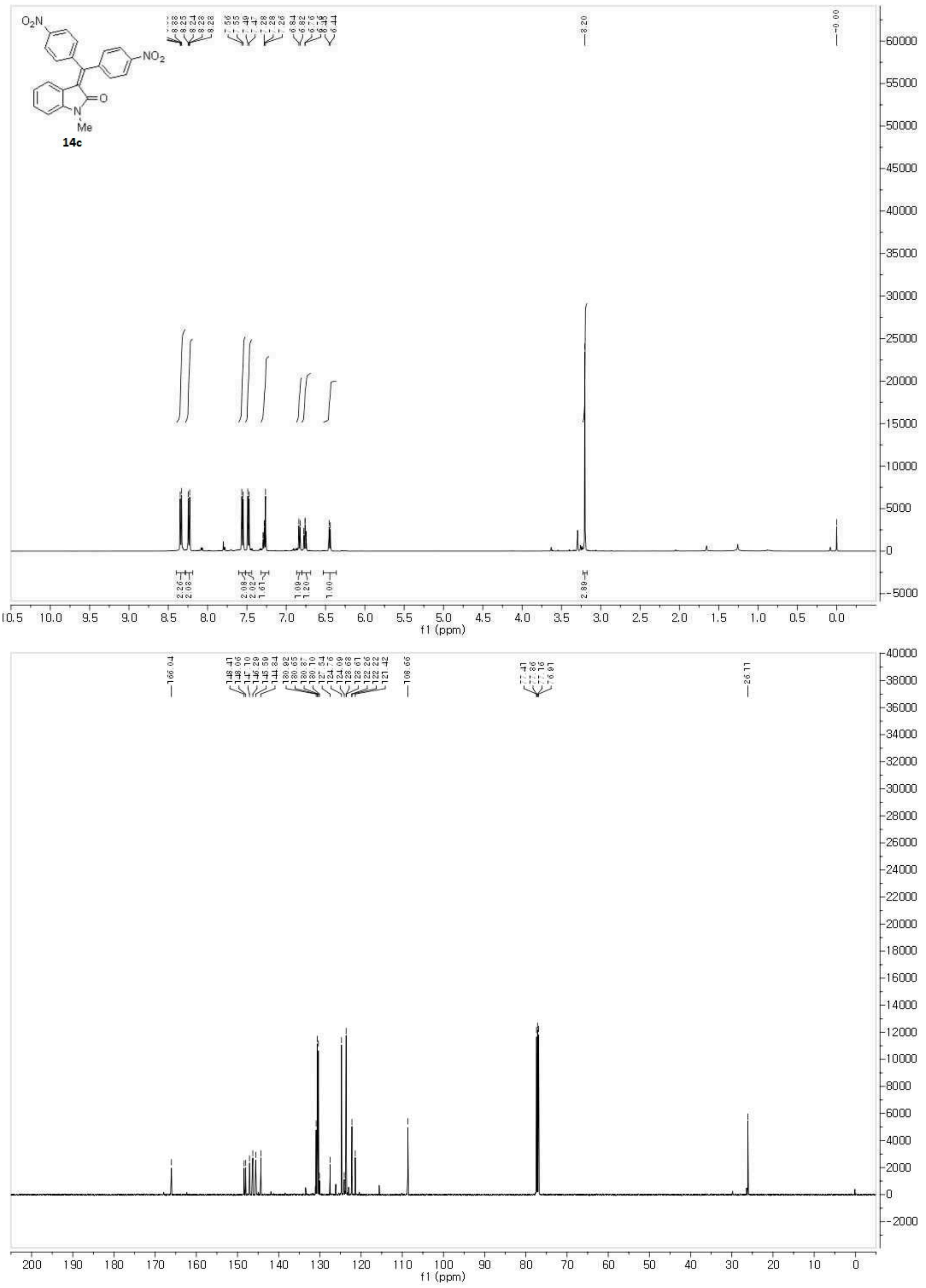

Figure S8. ^{1}H and ^{13}C spectrum of 3-(bis(4-nitrophenyl)methylene)-1-methylindolin-2-one (**14c**).

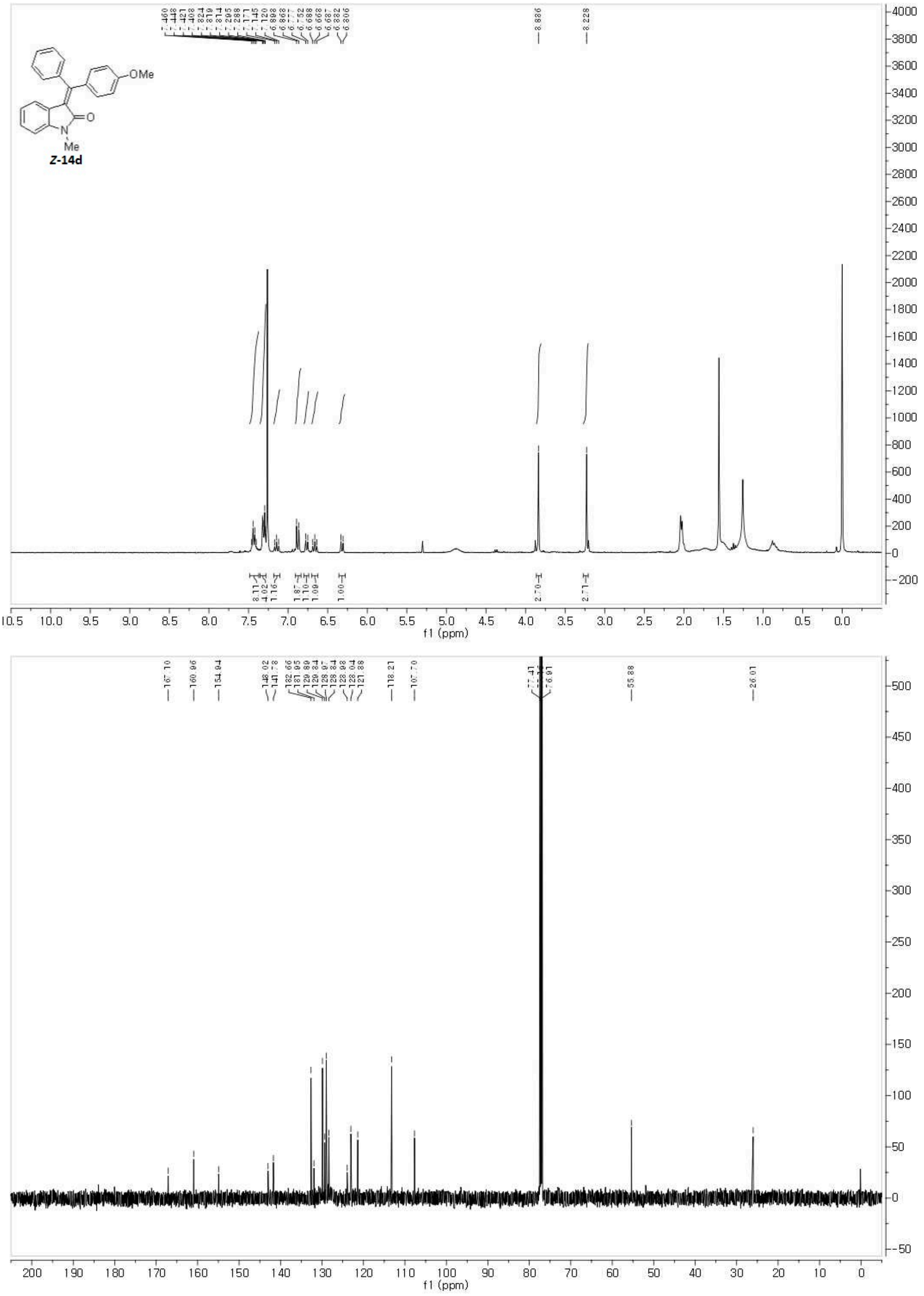

Figure S9. ^{1}H and ^{13}C spectrum of (*Z*)-3-((4-methoxyphenyl)(phenyl)methylene)-1-methylindolin-2-one (***Z*-14d**).

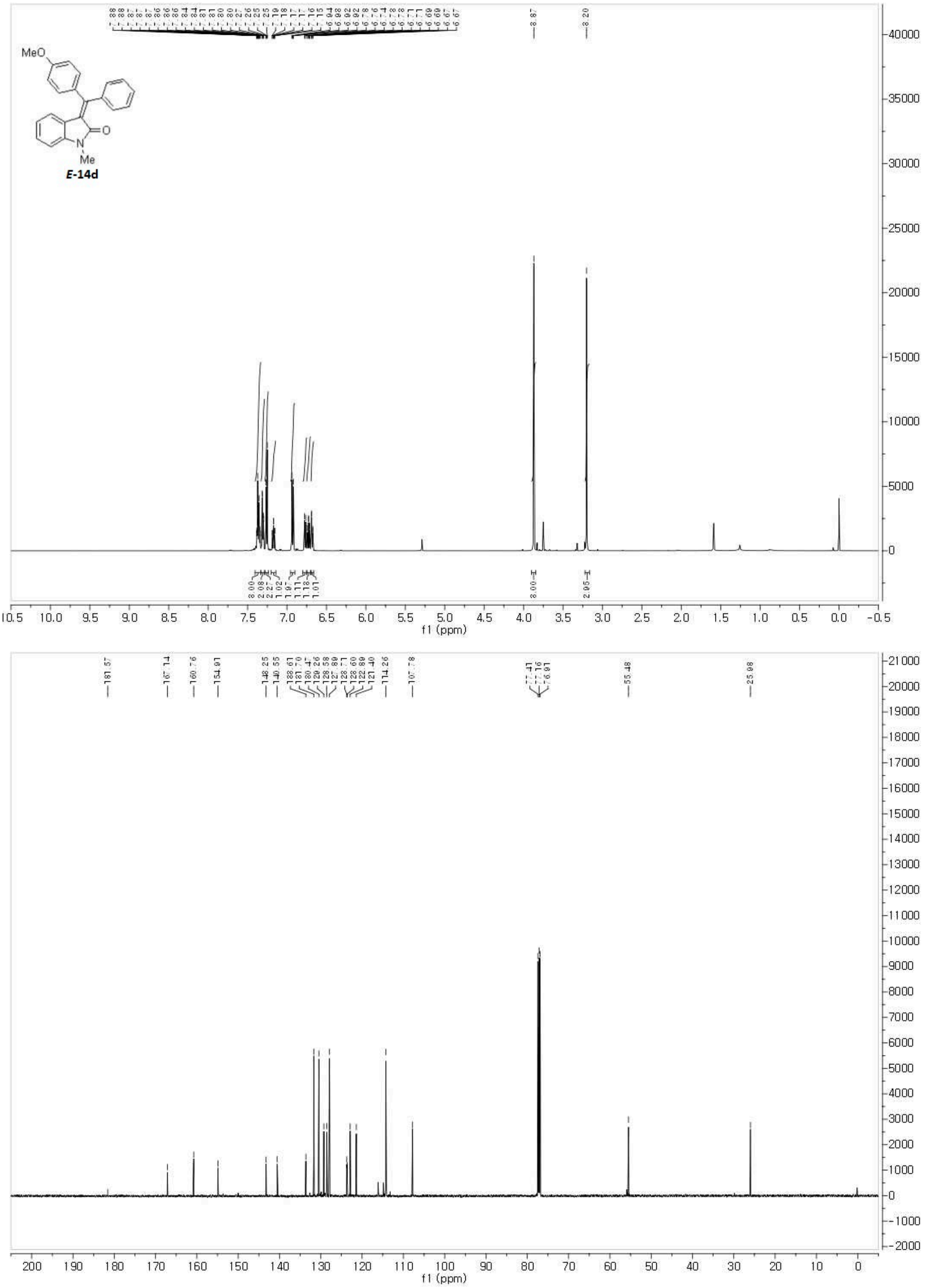

Figure S10. ^{1}H and ^{13}C spectrum of (*E*)-3-((4-methoxyphenyl)(phenyl)methylene)-1-methylindolin-2-one (***E*-14d**).

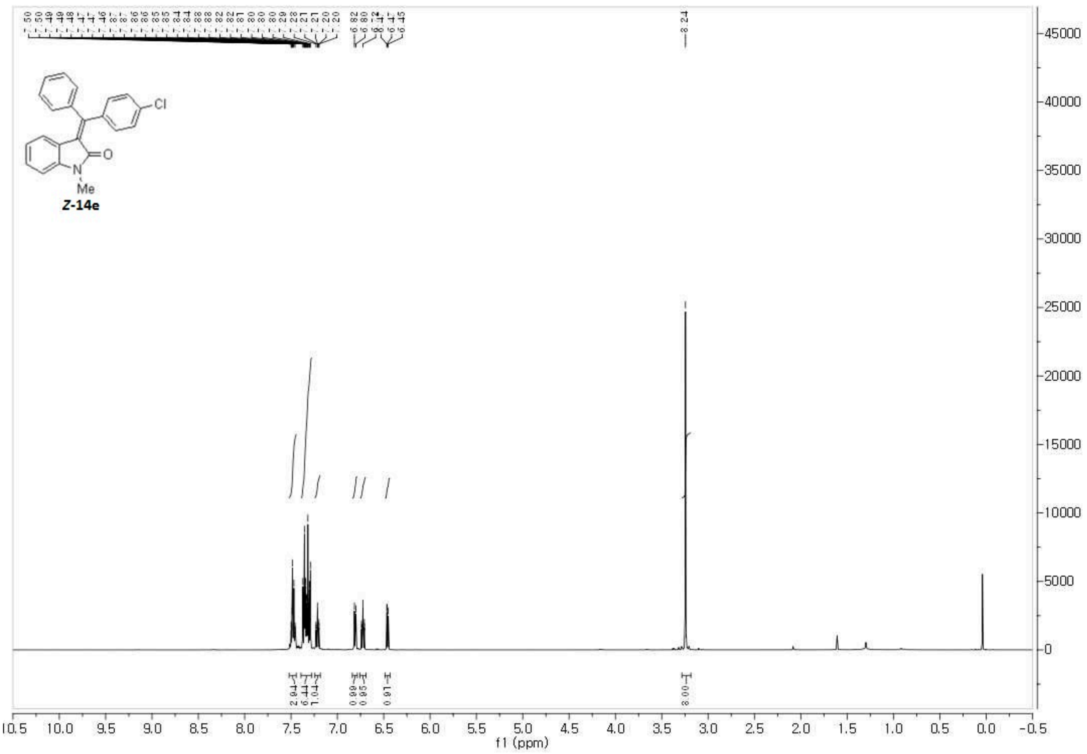

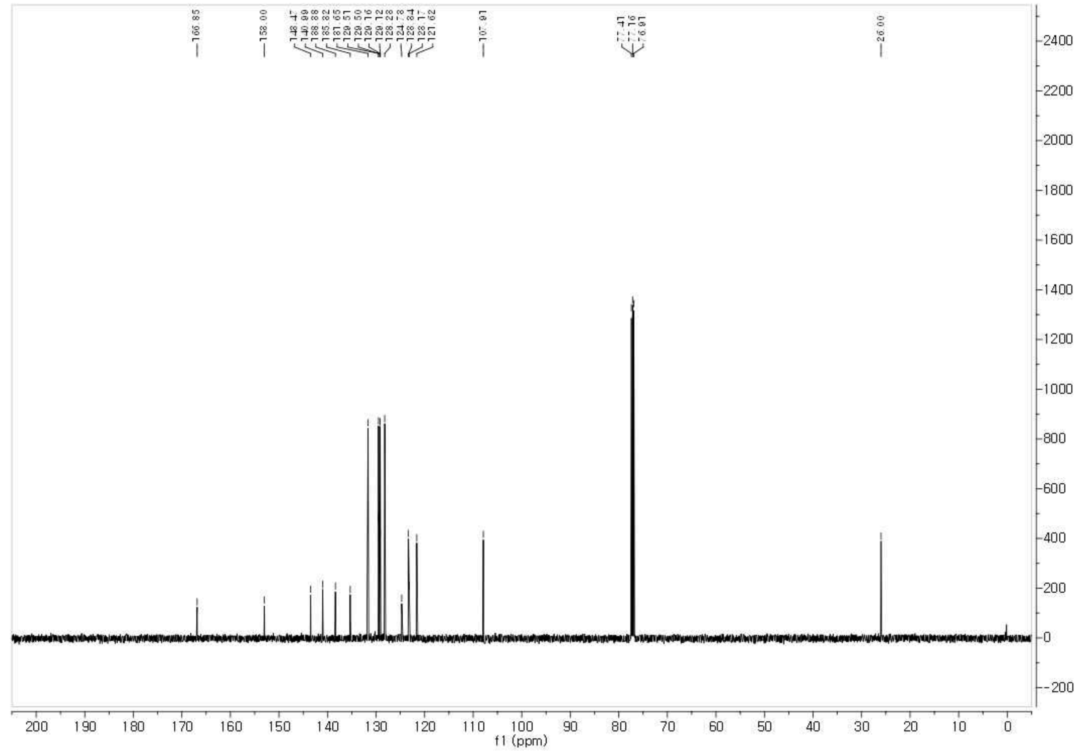

Figure S11. ^{1}H and ^{13}C spectrum of (*Z*)-3-((4-chlorophenyl)(phenyl)methylene)-1-methylindolin-2-one (***Z*-14e**).

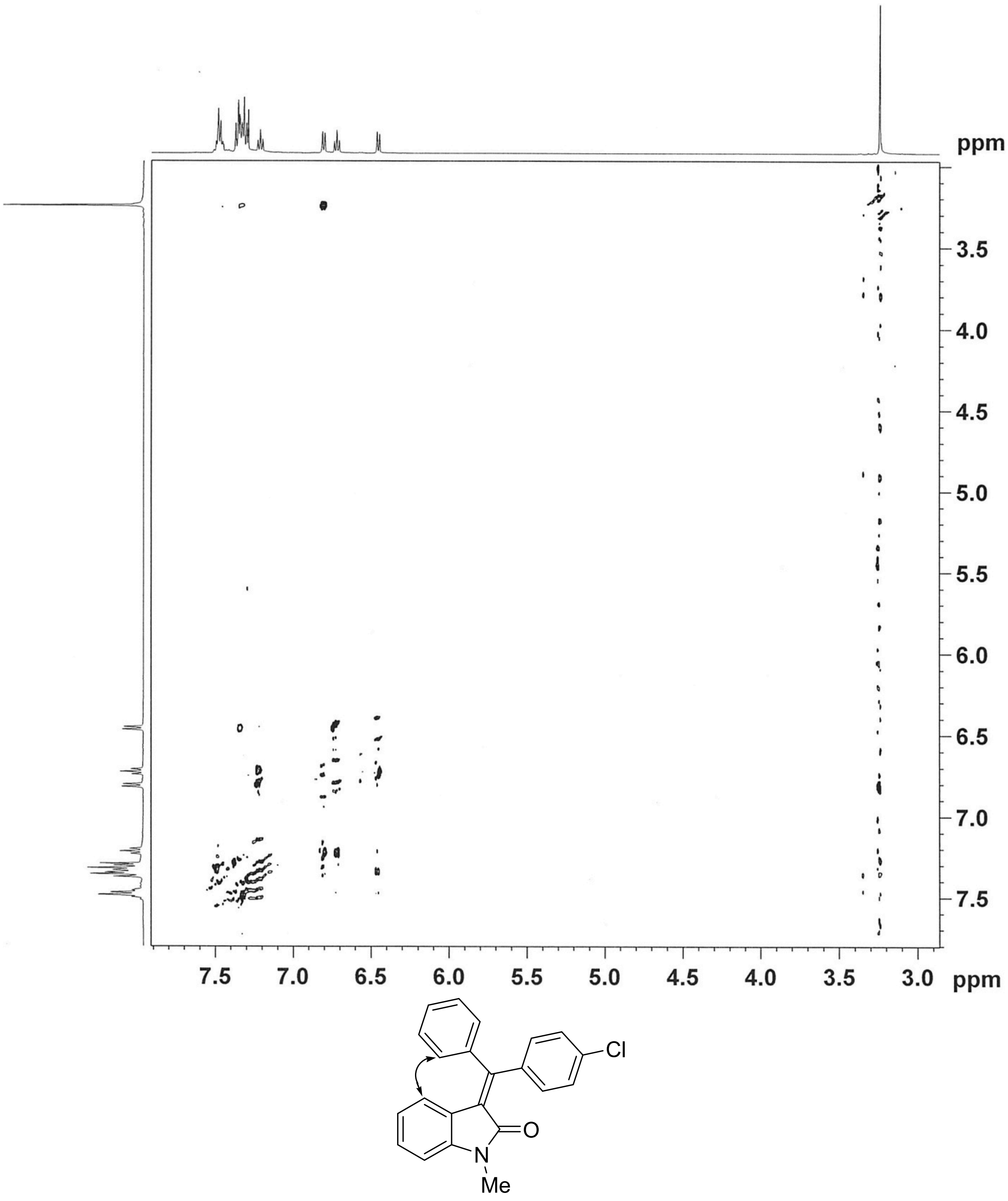

Figure S12. ROESY spectrum of ***Z*-14e**.

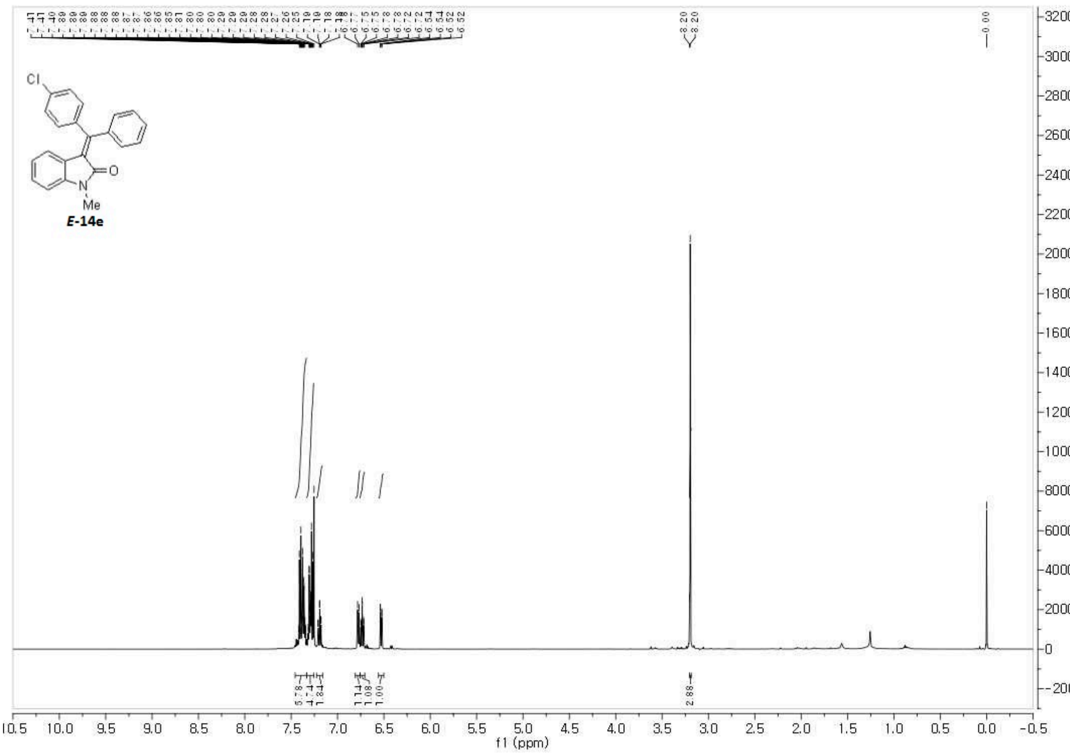

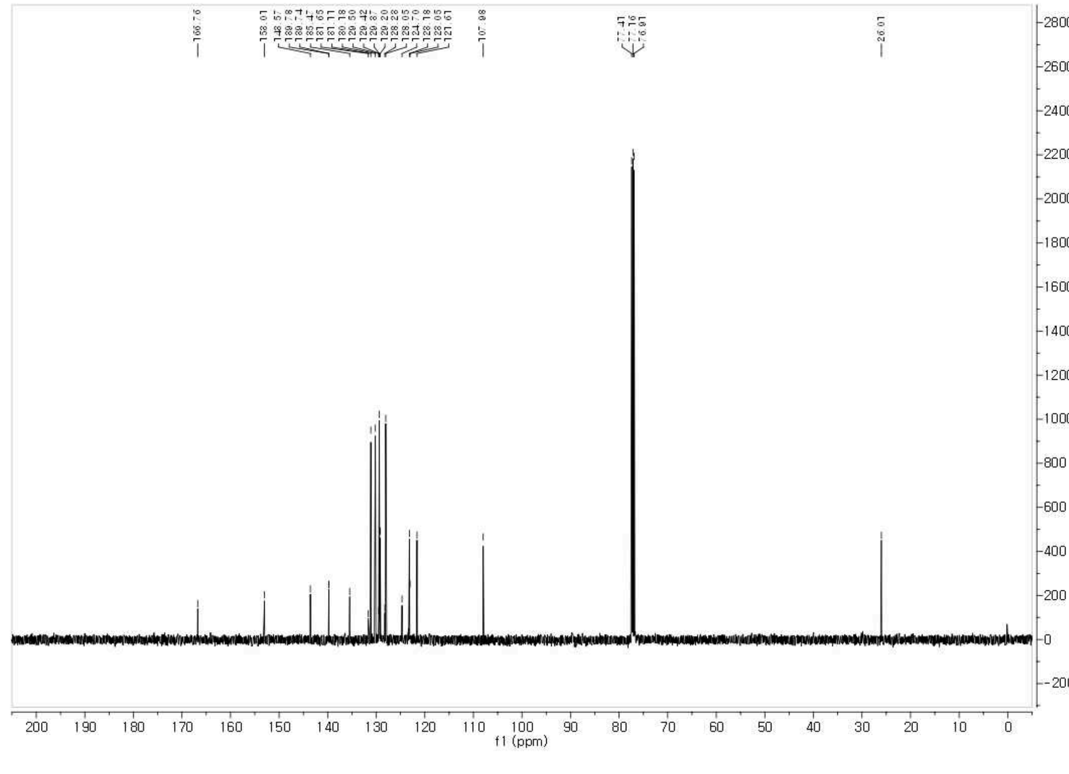

Figure S13. ^{1}H and ^{13}C spectrum of (*E*)-3-((4-chlorophenyl)(phenyl)methylene)-1-methylindolin-2-one (***E*-14e**).

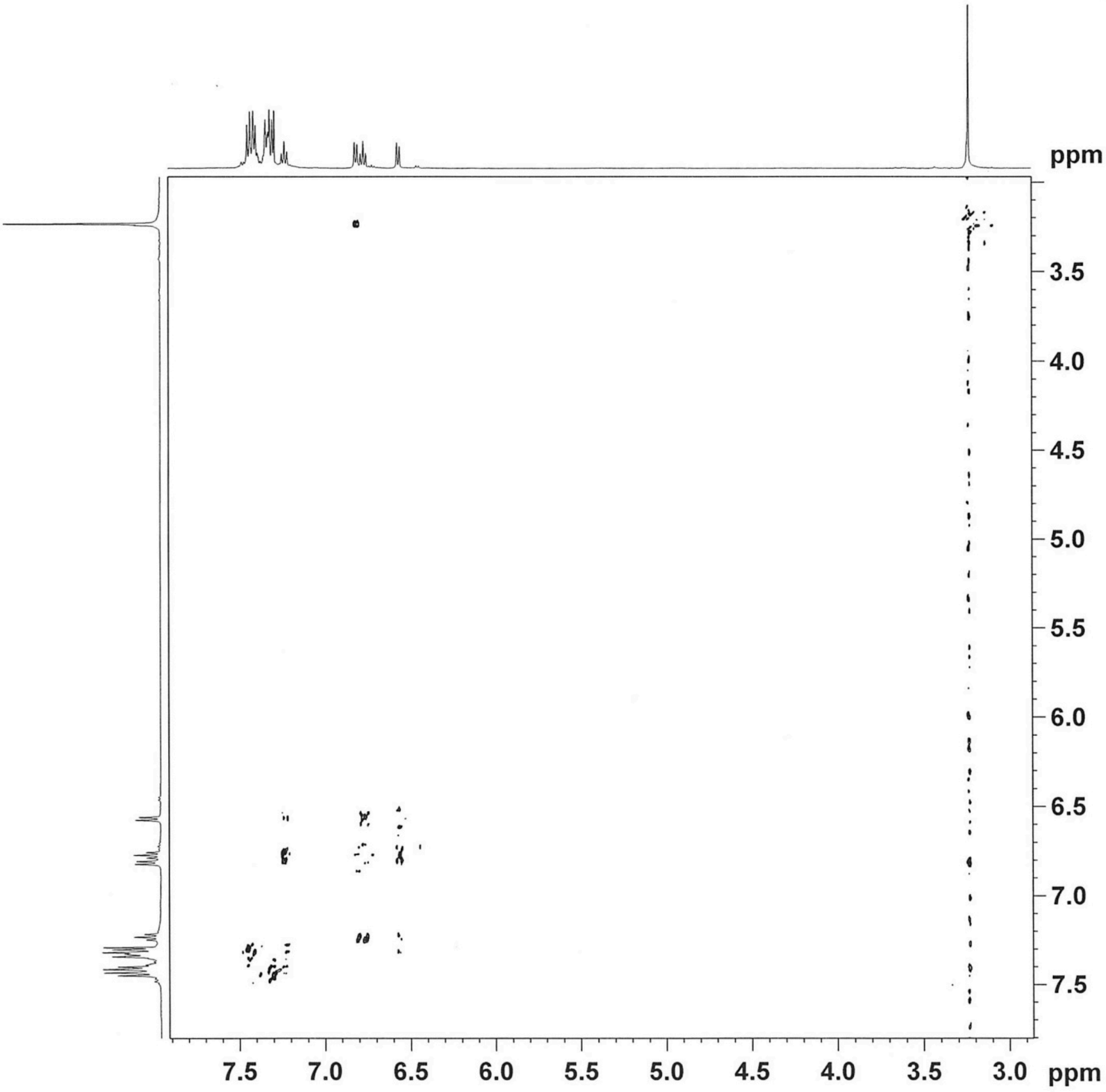

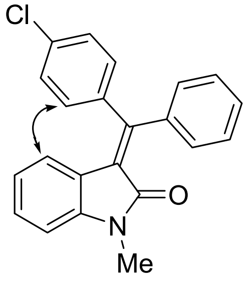

Figure S14. ROESY spectrum of ***E*-14e**.

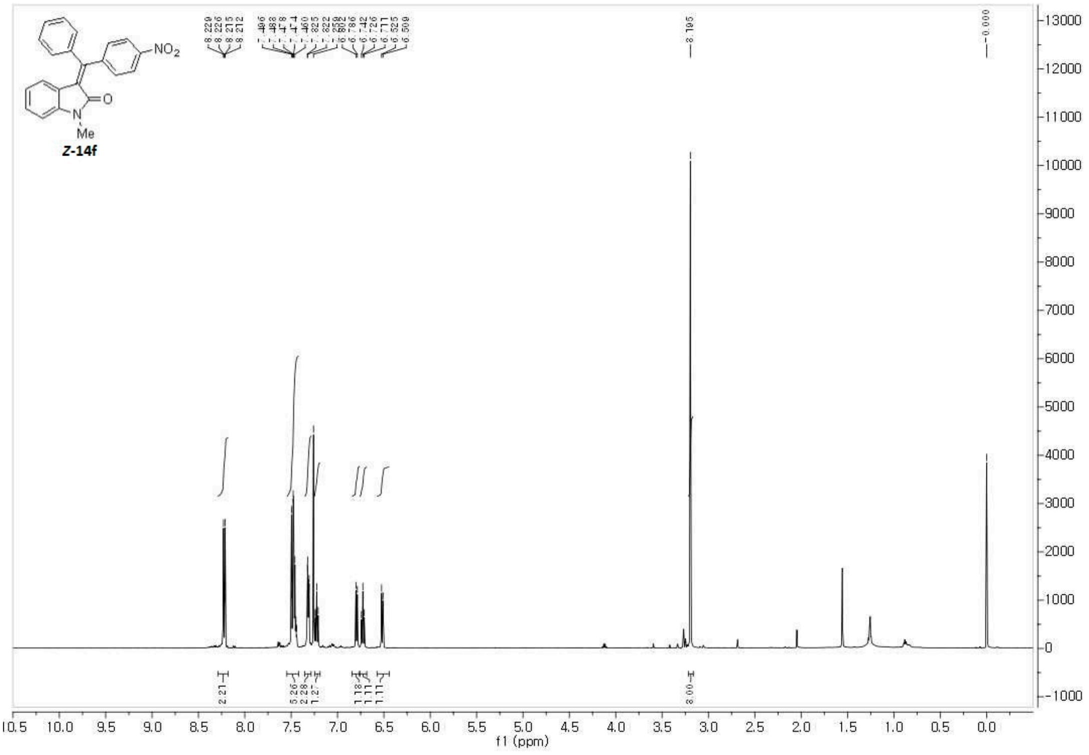

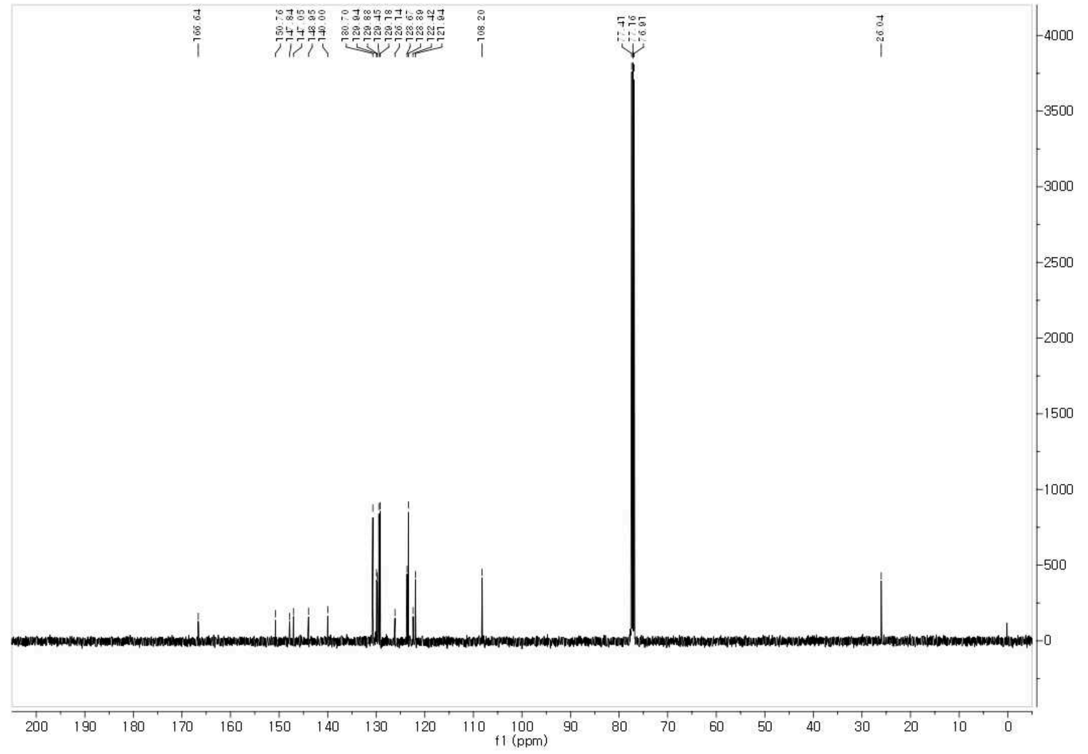

Figure S15. ^{1}H and ^{13}C spectrum of (*Z*)-1-methyl-3-((4-nitrophenyl)(phenyl)methylene) indolin-2-one (***Z*-14f**).

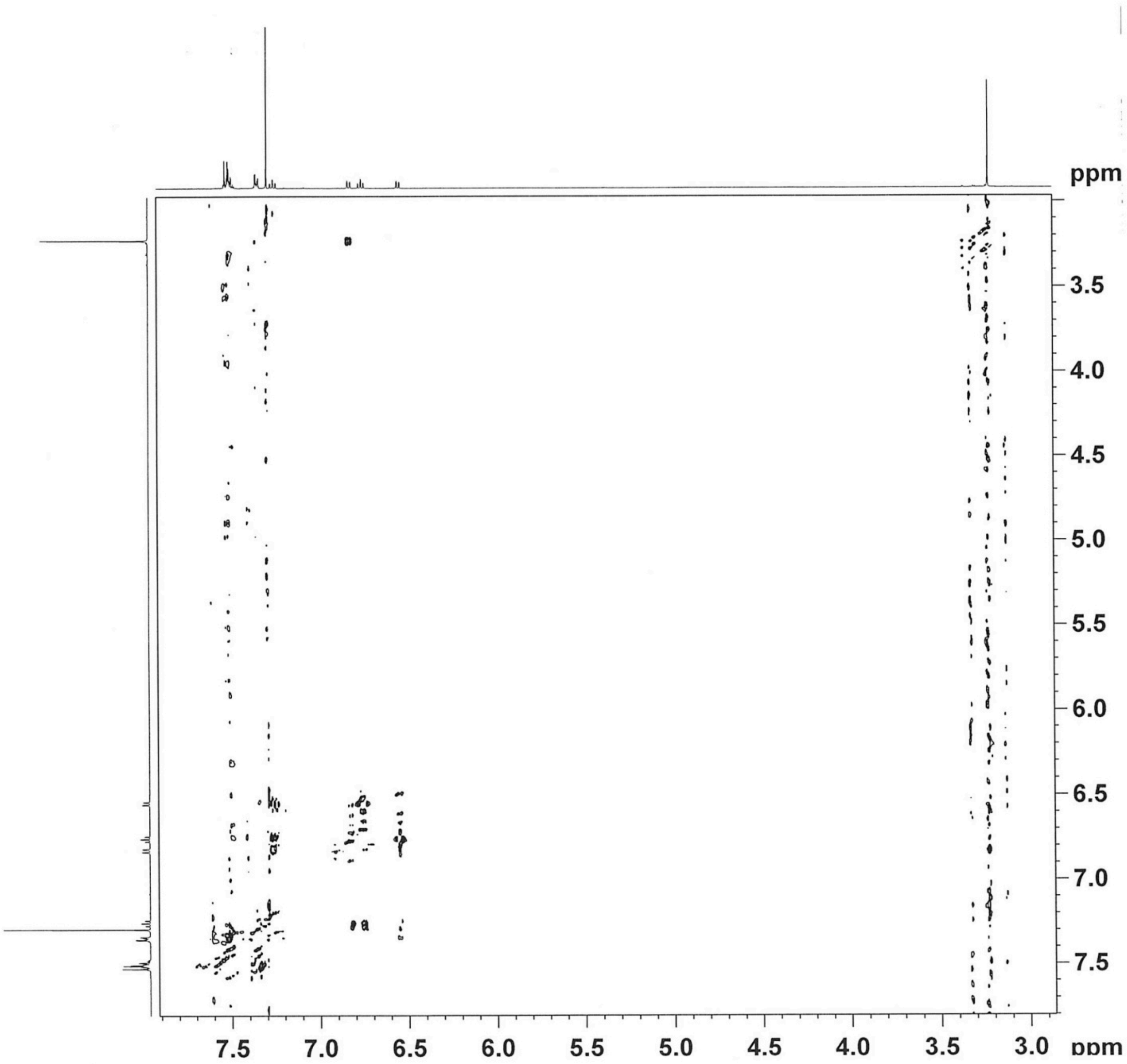

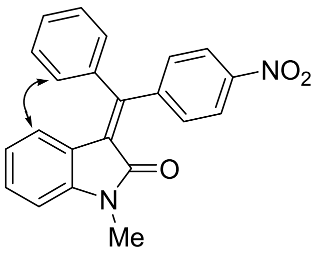

Figure S16. ROESY spectrum of ***Z*-14f**.

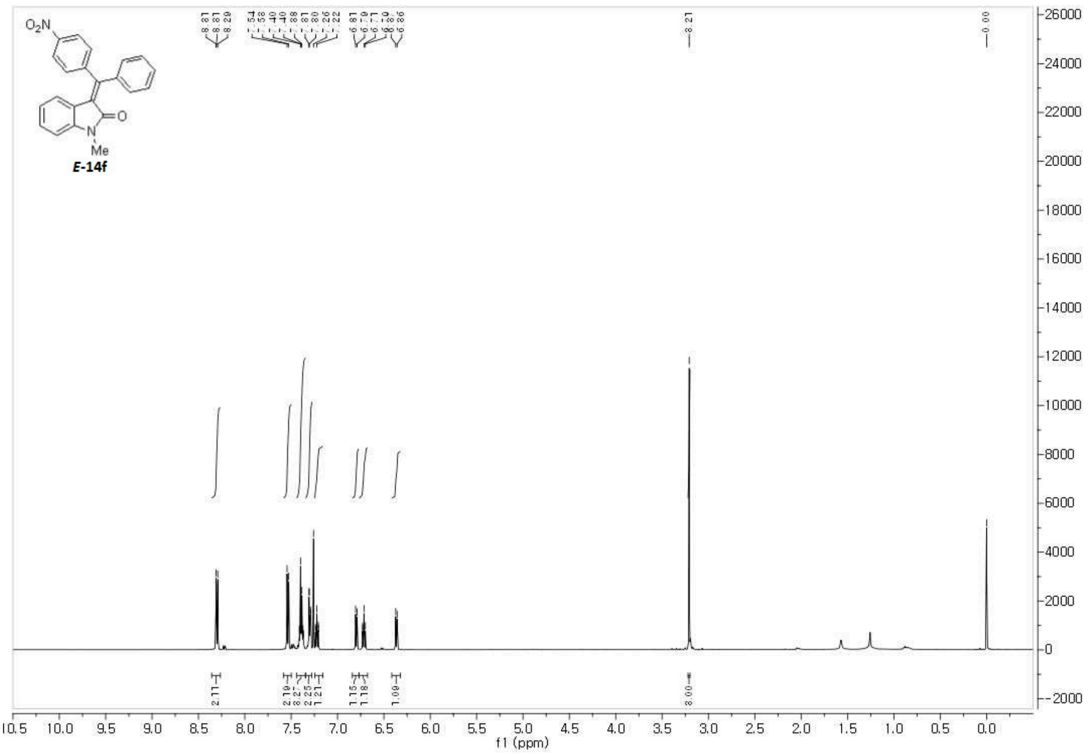

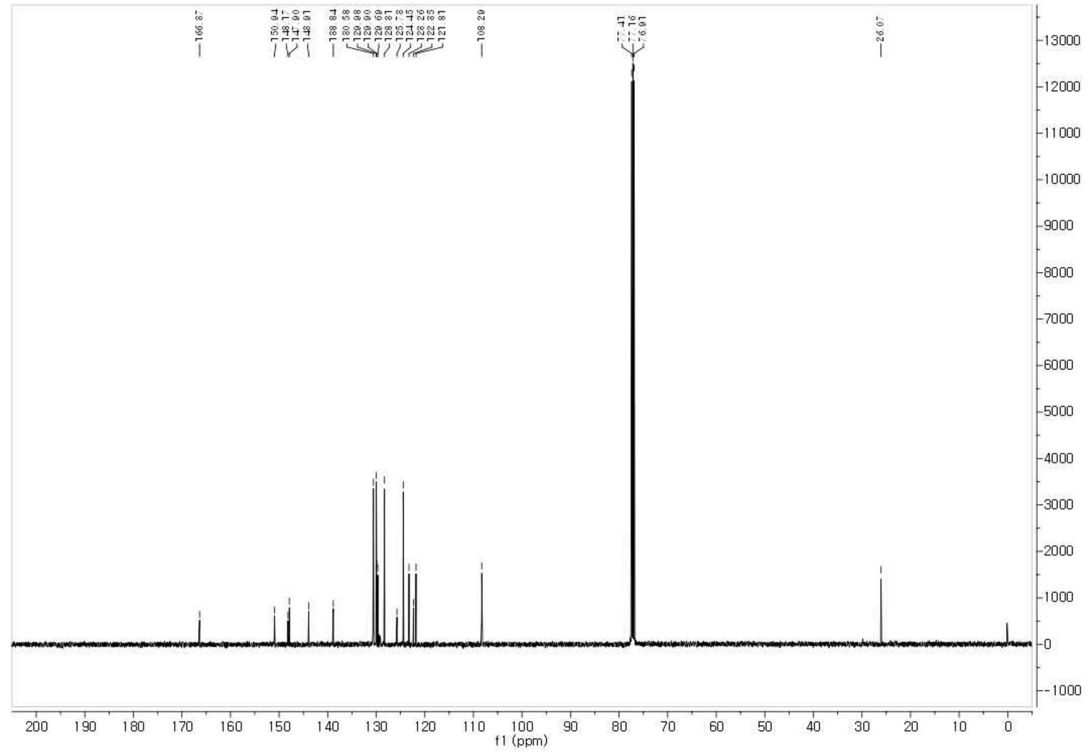

Figure S17. 1H and 13C spectrum of (*E*)-1-methyl-3-((4-nitrophenyl)(phenyl)methylene) indolin-2-one (***E*-14f**).
